# HuR-mediated regulation of mTOR mRNA stability promotes the commitment of satellite cells towards myogenesis

**DOI:** 10.1038/s41419-026-08759-1

**Published:** 2026-04-21

**Authors:** Anne-Marie K. Tremblay, Brenda Janice Sánchez, Bianca Colalillo, Souad Mubaid, Jason Sadek, Xian Jin Lian, Andrea Diaz Gaxiola, Sergio Di Marco, Imed-Eddine Gallouzi

**Affiliations:** 1 Department of Biochemistry, McGill University, Montreal, QC Canada; 2 Rosalind & Morris Goodman Cancer Institute, McGill University, Montreal, QC Canada; 3 KAUST Smart-Health Initiative (KSHI) and Biological and Environmental Science and Engineering (BESE) Division, King Abdullah University of Science and Technology (KAUST), Jeddah, Saudi Arabia

**Keywords:** Differentiation, RNA metabolism

## Abstract

The RNA-binding protein HuR has been shown to promote the differentiation of cultured muscle cells into muscle fibers. HuR mediates this process by differentially regulating, at different stages of this process, mRNA targets encoding pro-myogenic factors. Despite these advancements, the role of HuR, in vivo, at various stages of the myogenic process and its impact on muscle formation and function remain elusive. Towards this end, we used the Myf5-Cre loxP system to knock out HuR at a stage where muscle precursor cells (satellite cells, SCs) commit to myogenesis. Using these mice, we found that the muscle-specific depletion of HuR impairs, physiologically, its formation during embryogenesis and in response to injury. These mice exhibited smaller skeletal muscles and reduced exercise endurance. We demonstrate, using these mice, that this effect is due, in part, to the HuR-mediated regulation of mTOR (mechanistic target of rapamycin) mRNA expression. Using primary and cultured muscle cells, we show that HuR associates with this message, regulating its stability. In doing so, HuR facilitates the commitment of satellite cells toward myogenesis, thus preventing their transdifferentiation toward adipogenesis. These findings thus identify HuR as a master regulator of SCs' commitment to myogenesis and uncover a potential target for manipulating muscle myogenic capacity in both normal and pathological conditions.

## Introduction

Skeletal muscle, an essential organ constituting 40% of our total body mass, is required for locomotion, breathing, posture, metabolism, and body homeostasis [1]. The ability of a muscle to regulate its size and metabolic properties allows it to adapt to environmental challenges, such as exercise and injury [2]. During embryogenesis, there are two main stages that dictate the formation of muscle fibers [3–5]. During the first stage, which lasts from E8 to E14.5, progenitor cells, which are bipotent and capable of committing to either myogenesis (muscle fiber formation) or adipogenesis (formation of brown adipocytes), commit to the myogenic lineage, resulting in the foundation of muscle limbs [6, 7]. The second phase, which lasts from E14.5 to E16.5, involves building an overlay of myofibers required for the formation of functional muscle [8, 9]. Following the first 3 weeks postpartum, a fraction of precursor cells, named satellite cells (SC), located between the myofiber plasma membrane and the basal lamina, enter mitotic quiescence [2, 4, 5, 10]. These cells serve as the muscle stem cell pool required for post-natal myogenesis, referred to as muscle regeneration, which occurs in response to injury [8, 11].

The formation of skeletal muscle during embryonic development and in response to injury, also known as myogenesis, is a tightly regulated process [11, 12]. Myogenesis requires the coordinated interplay of several molecular mechanisms that mediate the various steps in this complex process. These steps, which include muscle cell commitment, proliferation, cell cycle withdrawal, and terminal differentiation into multinucleated myofibers, are all modulated by myogenic regulatory factors (MRFs) [5, 9]. These MRFs, including Myogenic Factor 5 (Myf5), Myogenic Differentiation antigen (MyoD), and Myogenin, are transcription factors that are sequentially activated during each stage of the myogenic process [9, 13]. The function of these MRFs is dictated by their temporal expression during myogenesis [14, 15]. Indeed, while the expression of Myf5 begins at E8.0 (at a point where muscle precursor cells become committed to the myogenic lineage, forming myoblasts), MyoD is expressed two days later (E10.5), when muscle progenitor cells start to fuse to give rise to muscle limbs [9, 16–18]. Therefore, the molecular mechanisms regulating the temporal expression of these factors play a prominent role in mediating the development and function of skeletal muscle.

Although the importance of mechanisms regulating the transcription of MRFs during myogenesis is well-established [2, 5, 12], several groups, including ours, have shown that the post-transcriptional regulation of MRF expression is also required for the proper formation and function of muscle fibers [13, 19–22]. These regulatory mechanisms, which regulate the splicing, localization, stability, and translation of mRNAs, play prominent roles in determining, in a spatial and temporal manner, the fate of muscle cells. The RNA-binding protein (RBP) Elavl1, also known as Human antigen R (HuR), is well-known to affect various cellular functions, including myogenesis through interactions with its mRNA targets and other trans-activating factors such as miRNAs and RBPs [19, 22–35]. Our laboratory and others have previously shown, in cellulo, the role of HuR during myoblast differentiation. During the earlier stages, HuR promotes myogenesis by inducing the translation of the alarmin high mobility group box 1 (HMGB1) mRNA [32] and by decreasing the stability of the nucleophosmin (NPM) mRNA [19]. At later steps of myogenesis, when myoblasts begin their differentiation and fusion to form fibers (myotubes), HuR associates with and stabilizes pro-myogenic mRNAs encoding promoters of muscle fiber formation such as MyoD, Myogenin, and p21^cip1^ [36–40]. While these data establish that HuR may be required for the commitment of muscle cells to myogenesis, whether this is the case in vivo remains to be established.

We recently generated an *Elavl1* muscle-specific knockout (muHuR-KO) mouse to investigate the in vivo role of HuR in muscle formation and muscle physiology [31]. These animals were generated by breeding mice carrying the *Elavl1*^*fl/fl*^ allele with mice expressing Cre recombinase under the control of the *MyoD* promoter (activated during embryogenesis as early as day E10.5). While these muHuR-KO mice were perfectly healthy with properly developed and functional skeletal muscles, we observed that they have higher exercise endurance associated with enhanced levels of fast-twitch oxidative type I fibers [31]. Although these findings demonstrated the importance of HuR in muscle physiology, it did not, to our surprise, provide any indication that HuR is required for the early steps of myogenesis in vivo [11, 19, 31–33, 37, 39–41]. This is likely because the depletion of HuR in these mice occurs at a time when muscle cells have already committed to the process. These observations, however, raised the possibility that during development and/or in response to muscle injury, HuR functions, in a temporal manner, to regulate the expression of mRNAs that mediate, in a coordinated fashion, the various stages of myogenesis.

To address this possibility, in this study, we explored the role of HuR, in vivo, at a stage of the myogenic program when the commitment of SCs is being established. Using a Myf5-driven Cre-LoxP system we show that knocking out HuR affected the commitment of myoblasts to the myogenic process, resulting in the decreased development of muscle. Moreover, we found that the Myf5-Cre-mediated depletion of HuR leads to muscles with reduced endurance capacity and impaired regeneration. By performing High-Throughput RNA sequencing experiments, we show that this phenotype is mediated, in part, by the altered expression of the mTOR mRNA. We established that HuR binds to the mTOR mRNA and regulates its stability in muscle cells. Mechanistically, we show that HuR, through the regulated expression of mTOR, promotes the formation/regeneration of skeletal muscle by controlling myogenic determination. Collectively, our work shows that HuR regulates, in a temporal manner, the formation and function of skeletal muscle by promoting the commitment of muscle precursor cells towards the myogenic population.

## Results

### The genetic ablation of HuR (Elavl1) during the determination phase of myogenesis negatively impacts muscle mass and function

**Fig. 1:**
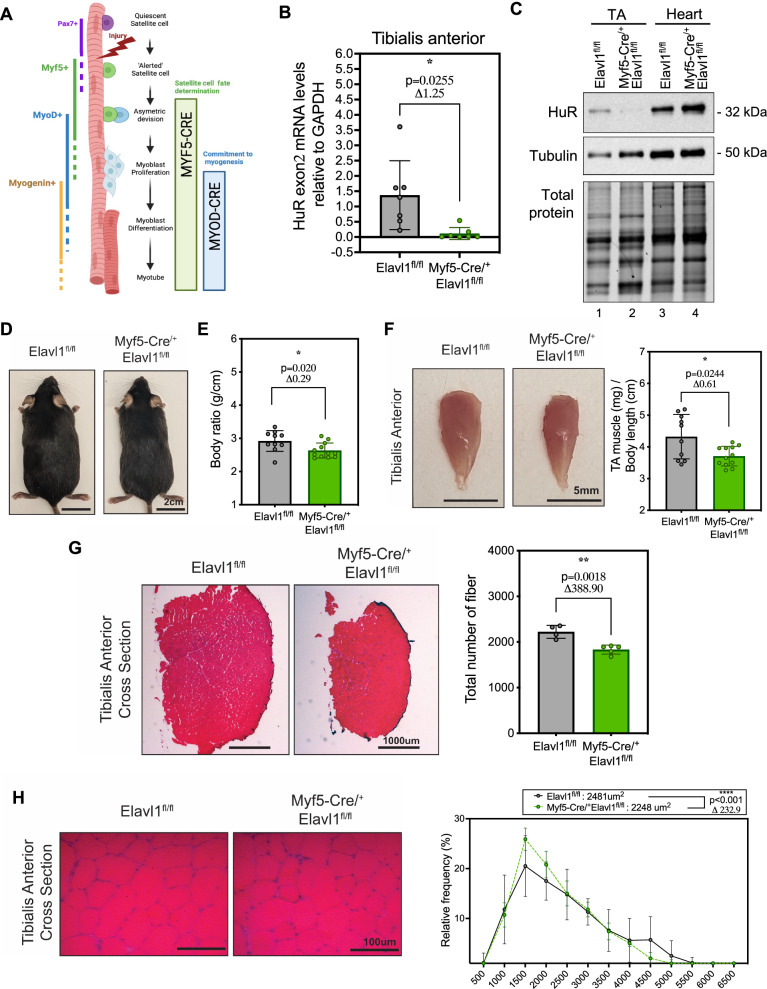
Knocking out HuR in pre-committed myogenic precursor cells affects muscle development. **A** Scheme demonstrating the steps involved in myogenesis. For the Myf5-Cre^/+^Elavl1^fl/fl^ mouse model, Cre recombinase, expressed under the control of the Myf5 promoter, is active during satellite cell myogenic determination. The scheme was generated using Biorender. **B** Validation of the knockout of HuR in Myf5-Cre^/+^Elavl1^fl/fl^ mice was assessed by quantifying the levels of exon 2 by RT-qPCR. **C** Representative western blot analysis of HuR protein levels in skeletal muscle (Tibialis Anterior, TA) and cardiac muscle (Heart, H) showing the specificity of HuR depletion to skeletal muscles. **D** Representative picture of 2-month-old male Myf5-Cre^/+^Elavl1^fl/fl^ mice compared to the control Elavl1^fl/fl^. Scale bar = 2 cm. **E** Body ratio of Myf5-Cre^/+^Elavl1^fl/fl^ mice. Body ratio was measured as the body weight (in grams) divided by body length (in centimeters). **F** Left: Image of the tibialis anterior muscle obtained from Myf5-Cre^/+^Elavl1^fl/fl^ mice. Right: quantification of muscle mass normalized to body. Scale bar = 5 mm. **G** Left: Picture of H&E-stained full cross-section of the tibialis anterior muscle of Myf5-Cre^/+^Elavl1^fl/fl^ mice. Right: Quantification of the total number of fibers (using ImageJ). Scale bar = 1000 μm. **H** Left: A zoomed image of H&E-stained tibialis anterior cross-section muscle of Myf5-Cre^/+^Elavl1^fl/fl^ mice. Right: Quantification, using ImageJ, of the average cross-sectional area of muscle fibers. Scale bar = 100 μm. At least 200 myofibers were measured for CSA analysis from *n* = 5 mice per group. **B**, **E**–**G** Each point in the scatter dot plots represents an individual animal. The columns on the plots represent the mean with standard deviation and unpaired *t* test (<0.05 = *, <0.01 = **, <0.0001 = ****). **B**, **F** Welch’s *t* test was used. See also Fig. S1.

To investigate the role of HuR on the commitment of SCs towards myogenesis and, furthermore, the subsequent formation/function of skeletal muscle, we used the Cre-LoxP strategy [31] to generate a new mouse model (Myf5-Cre^/+^Elavl1^fl/fl^) that is characterized by the Myf5-Cre driven knockout of HuR during the early stage of the myogenic program (Fig. 1A) [31]. Knockout of HuR under these conditions was confirmed by qPCR as well as western blot analysis in several skeletal muscles, including the TA, gastrocnemius, soleus, extensor digitorum longus, and peroneus (Fig. 1B, C and S1a–d). The knockout of HuR was furthermore specific to skeletal muscle since its expression was not affected in cardiac muscle (Fig. 1C).

Although the Myf5-Cre driven HuR knockout mice were not physically (Fig. 1D) or developmentally (Fig. S1e) different than their control counterparts, we observed that they were, nonetheless, characterized by smaller body weights (Fig. S1f) and body ratios (Fig. 1E). The Myf5-Cre^/+^Elavl1^fl/fl^ mice indeed have lower normalized body weights, indicating that they are slimmer rather than smaller. We next investigated if the decrease in body weight observed in the muscle-specific HuR knockout mice is due to an effect on the integrity of skeletal muscle. We observed that the mass of the TA muscle in these HuR-KO mice was 14.2% less than the wild-type controls (Fig. 1F). We additionally showed similar significant reductions in other skeletal muscles, including the soleus and the gastrocnemius as well as trends in the reductions of other muscles including the extensor digitorum longus and the peroneus muscles (Fig. S1g–j). Since we did not detect significant changes in the mass of other tissues, including the heart or white adipose tissue (Fig. S1k, l), we attributed the overall reduction in body weight of the Myf5-Cre^/+^Elavl1^fl/fl^ mice to decreases in skeletal muscle mass. To assess the impact of the depletion of HuR on muscle formation and fiber integrity, we next measured the total number and cross-sectional area of fibers in the TA. We show that Myf5-Cre^/+^Elavl1^fl/fl^ tibialis anterior muscle has a lower total number of fibers (Fig. 1G) and a smaller cross-sectional area than their control counterparts (Figs. 1H and S1m). The total surface area of the fibers, when normalized to body length, was also lower (Fig. S1n). Overall, our results indicate that the cumulative effect of the reduced number of fibers and their reduced size likely contributes to the smaller muscle mass observed in the Myf5-Cre driven HuR knockout mice.

**Fig. 2:**
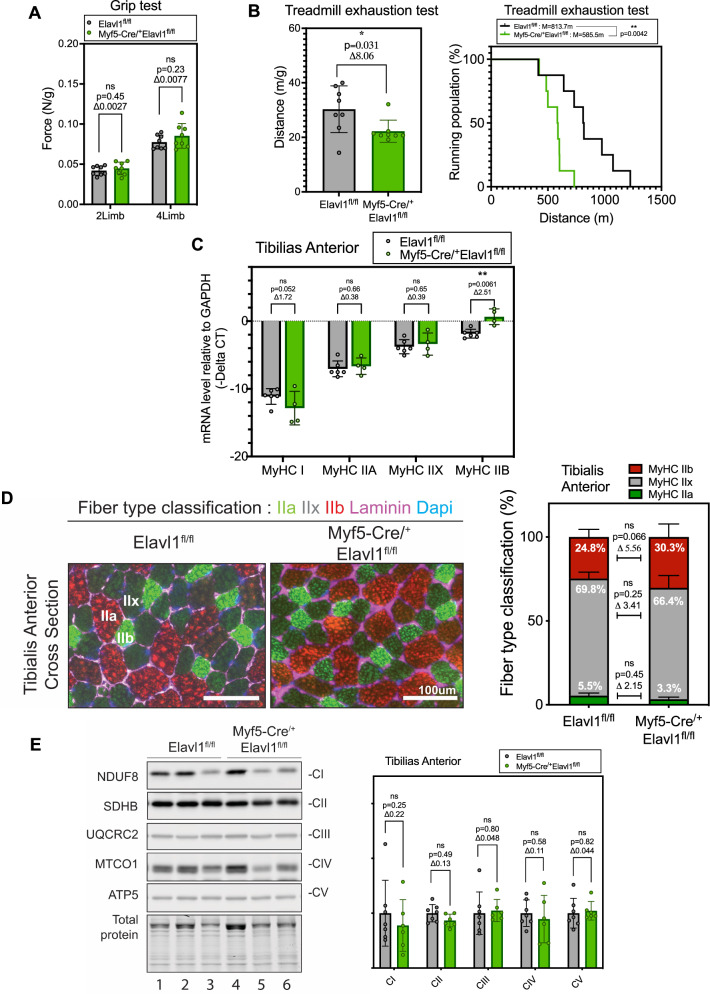
Myf5-Cre induced knockout of HuR negatively impacts muscle endurance capacity. **A** The strength of the two- and four-limb muscle grip of Elavl1^fl/fl^ and Myf5-cre^/+^Elavl1^fl/fl^ mice. Force in Newton (N) was normalized to body weight (g). **B** Left: Treadmill exhaustion test represented by percentage population running of Elavl1^fl/fl^ and Myf5-cre^/+^Elavl1^fl/fl^ mice. The statistics used were a Log-rank (Mantel–Cox) test. The percentage population running was done with *n* = 8 per group. Right: Treadmill exhaustion test measured by distance normalized to body weight of Elavl1^fl/fl^ and Myf5-cre^/+^Elavl1^fl/fl^ mice. **C** Myosin heavy chain type *I, IIa, IIx*, or *IIb* mRNA levels relative to *GAPDH* mRNA in the tibialis anterior muscle were measured by quantitative RT-PCR. **D** Left: Representative Immunofluorescence images of fiber-type classification in the tibialis anterior muscle of Elavl1^fl/fl^ and Myf5-cre^/+^Elavl1^fl/fl^ mice. Fiber-type classification was analyzed using staining for Myosin heavy chain type IIa (red), IIx (unstrained), or IIb (green). Laminin (purple) was used to identify the muscle fiber perimeter. Scale bar = 100 μm. Right: Quantification of fiber type classification was done using MuscleJ analysis. The statistics used were unpaired *t* tests performed between each fiber type. The fiber-type classification was done with *n* = 7 for Elavl1^fl/fl^ and *n* = 4 for Myf5-Cre^/+^Elavl1^fl/fl^ mice. **E** Representative western blot analysis of the levels of oxidative phosphorylation (OXPHOS) complex subunits (I–V) in the tibialis anterior muscle of Elavl1^fl/fl^ and Myf5-cre^/+^Elavl1^fl/fl^ mice (*n* = 3 mice per group). Quantification of each subunit in the complex was normalized to total protein and was done with *n* = 6 for Elavl1^fl/fl^ and *n* = 7 for Myf5-Cre^/+^Elavl1^fl/fl^ mice. **A–C**, **E** Each point in the scatter dot plots represents an individual animal. The columns on the plots represent the mean with standard deviation. The statistics used were unpaired *t* tests (<0.05 = *, <0.01 = **).

We have previously shown that MyoD-driven muscle-specific HuR knockout mice have functional muscles and are characterized by an enhanced endurance capacity (resulting from the increased formation of oxidative fibers) [31]. Since Myf5-Cre driven HuR knockout mice, unlike these mice, have smaller muscles, we questioned how a decrease in their size would affect their functionality. We, therefore, assessed the muscle function of Myf5-Cre^/+^ Elavl1^fl/fl^ mice by measuring both muscle force and endurance capacity. Although we did not observe any significant changes in muscle force (assessed by measuring the two-limb and four-limb muscle grip strength) (Fig. 2A), our data indicate that the Myf5-Cre, unlike the MyoD-Cre driven HuR knockout mice [31], exhibit a significant decrease in exercise endurance (Fig. 2B) when compared to the control group. This decrease was not due to an effect on fiber-type specification since we did not observe any significant changes in the expression levels of mRNAs encoding Type I or II myosin Heavy chains (Fig. 2C) or the composition of oxidative and glycolytic fibers in the tibialis anterior muscle (Fig. 2D). We further show that Myf5-Cre driven HuR depletion does not affect the oxidative capacity of these mice as determined by western blot analysis of the subunit of each complex (I–V) of the mitochondrial oxidative phosphorylation (OXPHOS) system (Fig. 2E). Together, these results indicate that the muscle-specific depletion of HuR at early stages of myogenesis (~E8.0) negatively impacts the endurance performance of these animals. This observed effect, however, is not due to changes in fiber-type specifications or due to a reduced capacity to produce ATP through the electron transport chain (Fig. 2D, E). Overall, the reduction in muscle mass and function in the Myf5-Cre^/+^Elavl1^fl/fl^ model, in contrast to our previously characterized MyoD-Cre^/+^Elavl1^fl/fl^ model [31], suggests that HuR plays a prominent role in determining the specification of myogenic precursor cells.

### HuR is required for the myogenic program during muscle regeneration

During muscle injury, satellite cells (SC) that were developed during embryogenesis and are located between the myofiber plasma membrane and the basal lamina serve as the muscle stem cell pool needed for the regeneration of the muscle tissues [10, 11]. These cells, upon injury to muscle, exit their quiescent state and enter the myogenic program in order to initiate the repair of the muscle [42]. We thus made use of the cardiotoxin-induced muscle injury model [43] to evaluate the importance of HuR on the injury-induced commitment of satellite cells to the myogenic program and the resulting regeneration of skeletal muscle.

As a first step, we validated the cardiotoxin-induced model of muscle regeneration using our control Elavl1^fl/fl^ mice by assessing muscle integrity at different days post-injury (dpi) with cardiotoxin (Fig. S2a). We observed that muscle damage is clear during the early stages of this process (3 days post-injury). Proper fiber formation, however, begins to occur several days later (7–21 days post-injury) as evidenced by clearance of necrotic tissue and myofiber growth (Fig. S2b), as well as the increased expression of myogenin, a pro-myogenic factor known to mediate muscle regeneration (Fig. S2c). Moreover, we observed an increase in HuR mRNA and protein levels during early muscle regeneration (Fig. S2d, e), further suggesting that HuR plays an important role in the initiation of muscle formation.

**Fig. 3:**
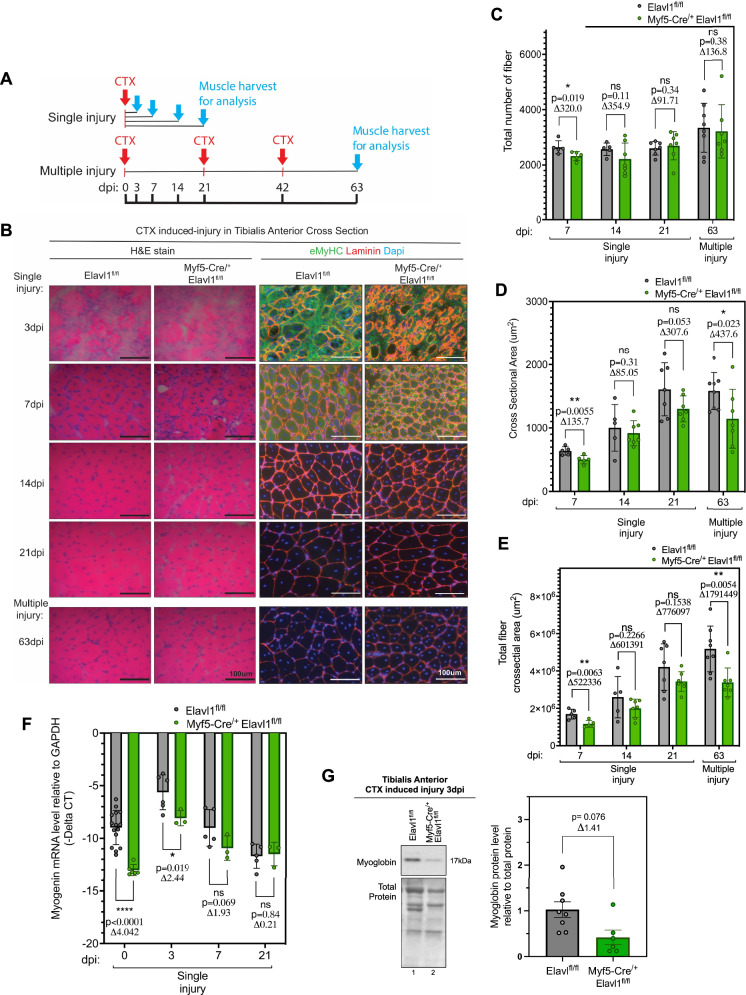
HuR is required in pre-committed myoblast for efficient muscle regeneration. **A** Representative scheme of cardiotoxin-induced injury for muscle regeneration experiment using Elavl1^fl/fl^ and Myf5-cre^/+^Elavl1^fl/fl^ mice. Single injury consisting of one injection of cardiotoxin (CTX, red arrow) followed by harvesting (blue arrow) at different days post-injury (dpi). Multiple injuries consisted of 3 injuries with 21-days intervals and with the muscle harvested 21 days after the last injection of cardiotoxin. **B** Left: Cross-sections of injured tibialis anterior muscles stained with H&E. Right: Cross-sections of injured tibialis anterior muscles labeled for newly formed fibers as assessed by immunofluorescence of embryonic myosin heavy chain (eMyHC, green). Staining for Laminin (red) was used for fiber perimeter and for nuclei by DAPI (blue). Scale bar = 100 μm. **C** Quantification of the total number of fibers at different time points during muscle regeneration. All fibers were measured for the total number of fibers analysis using OPEN-CSAM on ImageJ, followed by manual corrections. **D** Quantification of average cross-sectional area and **E** total cross-sectional area at different time points during muscle regeneration. All fibers were measured for CSA analysis using OPEN-CSAM on ImageJ, followed by manual corrections. **F** *Myogenin* mRNA levels relative to GAPDH were determined by RT-qPCR in tibialis anterior muscle lysis after cardiotoxin-induced muscle regeneration. Muscles at 0 dpi represent uninjured muscle. **C**–**G** Representative western blot analysis of the levels of Myoglobin in the tibialis anterior muscle of Elavl1^fl/fl^ and Myf5-cre^/+^Elavl1^fl/fl^ mice 3 day post-injury. Quantification of each mouse was normalized to total protein and was done with *n* = 8 for Elavl1^fl/fl^ and *n* = 6 for Myf5-Cre^/+^Elavl1^fl/fl^ mice. Each point in the scatter dot plots represents an individual animal. The columns on the plots represent the mean with standard deviation. The statistics used were unpaired *t* tests (<0.05 = *, <0.01 = **, <0.0001 = ****). See also Figs. S2 and S3.

To test the importance of HuR in this process, we assessed muscle fiber regeneration in Myf5-Cre^/+^Elavl1^fl/fl^ mice injured with cardiotoxin (Fig. 3A). We observed that the size and number of myofibers were significantly less (21% and 12%, respectively) than the control group at 7 days post-injury (Figs. 3B–E and S3a). A trend towards a decrease in the size and number of these fibers was maintained until 21 days post-injury (Fig. 3B–E). Our results, therefore, show that HuR affects the initiation of muscle formation in vivo and that this effect is dependent on the timing of its knockout during the myogenic program.

The decrease in the size and number of muscle fibers seen in the Myf5-Cre knockout mice could be due to a defect in the maturation of the muscle. We, therefore, performed immunofluorescence experiments assessing the expression of embryonic myosin heavy chain (eMyHC). eMyHC, a marker of muscle regeneration, is the first myosin heavy chain isoform to be expressed upon injury to muscle. The levels of eMyHC, however, decrease as myofiber mature giving way to the expression of MyHCs that dictate the specification and metabolic properties of muscle fibers [44]. Our results show that the presence of eMyHC during the different stages of regeneration is less in the knockout mice when compared to their control counterparts. Indeed, we observe a greater presence of eMyHC during the early stages of regeneration, 3- and 7-days post-injection (dpi) in the control group when compared to the knockout mice. The presence of eMyHC, however, although decreased, is nonetheless similar at 14 and 20 dpi in both control and knockout animals (Fig. 3B) indicating that although knocking out HuR delays the regeneration of muscle it does not prevent their maturation (as evidenced by the similar profile of Type 1 and 2 fibers in the muscle of these animals, Fig. 2C, D). These results, therefore, indicate that the reduction in myofiber size is not due to a delay in their maturation.

We next investigated whether the pool of quiescent satellite cells required for muscle regeneration was impaired. During regeneration, through asymmetric division of quiescent satellite cells, a portion of these dividing cells will commit to the myogenic program to form new fibers. However, a fraction of these cells will divide to replenish the pool of quiescent satellite cells that are needed for future subsequent injuries [45]. This portion of self-renewing satellite cells has been shown to be critical for proper muscle regeneration, as prolonged depletion of the quiescent pool will result in an insufficient number of satellite cells capable of committing to the muscle repair process [11]. We, therefore, performed multiple injuries with cardiotoxin to evaluate the maintenance of the quiescent satellite cell population during muscle regeneration. This experiment consisted of 3 subsequent cardiotoxin-induced injuries (with 21 days intervals between each injury) followed by the harvesting of muscle 21 days after the last injection of cardiotoxin (Fig. 3A). We observed, by performing this experiment, that the maturation of fibers (shown by the absence of eMyHC expression) was similar between the knockout and control mice (Fig. 3B). In addition, H&E images revealed no retention of necrotic or damaged myofibers in muscle (Fig. 3B) nor any difference in fiber number following multiple injuries between both groups (Fig. 3C). We did, however, determine that the average cross-sectional area of myofibers and total sectional area following multiple injuries (63 days) is significantly lower (28% decrease) in Myf5-Cre^/+^Elavl1^fl/fl^ mice when compared to their control counterparts (Fig. 3D, E). This reduction in CSA resembles what we observed 21 days post-injury due to a single injection with cardiotoxin, indicating that multiple injections with cardiotoxin did not enhance the effects seen with a single dose. As such, our results indicate that satellite cell self-renewal is not impaired in HuR knockout mice since the extent of the effects observed after multiple injuries is no different than what was observed after a single injury. These results, in addition to the fact that the basal number of quiescent satellite cells in uninjured muscle of both control and HuR knockout mice is similar (Fig. S3b), therefore suggest that the overall reduction in CSA is not due to alterations in the quiescent satellite cell pool but rather due to their lack of myogenic commitment during muscle regeneration.

Myogenic commitment and fusion of mononucleated cells into multinucleated fibers are defined by the presence of myogenic regulatory factors such as Myogenin. We, therefore, measured *Myogenin* mRNA levels to evaluate myogenic commitment and cell fusion in control and Myf5-Cre-driven HuR knockout mice. We detected a significant decrease (45% and 43% respectively) in *Myogenin* mRNA in untreated muscle and muscle harvested at 3 days post-injury in the Myf5-Cre driven knockout mice when compared to their control counterparts (Fig. 3F). The decreased expression of Myogenin likely contributed to the delayed regeneration of muscle fibers in the injured-HuR knockout mice as evidenced by the reduced levels of myoglobin (a well-accepted marker of mature muscle fibers) in these mice relative to their control counterparts (Fig. 3G). Our results, therefore, show that skeletal muscles in Myf5-Cre-driven HuR knockout mice exhibit a reduction in their regeneration efficiency following injury. This effect is likely due to the ability of HuR to regulate the commitment and the myogenic program of satellite cells.

### HuR promotes the expression of mTOR during myogenesis

**Fig. 4:**
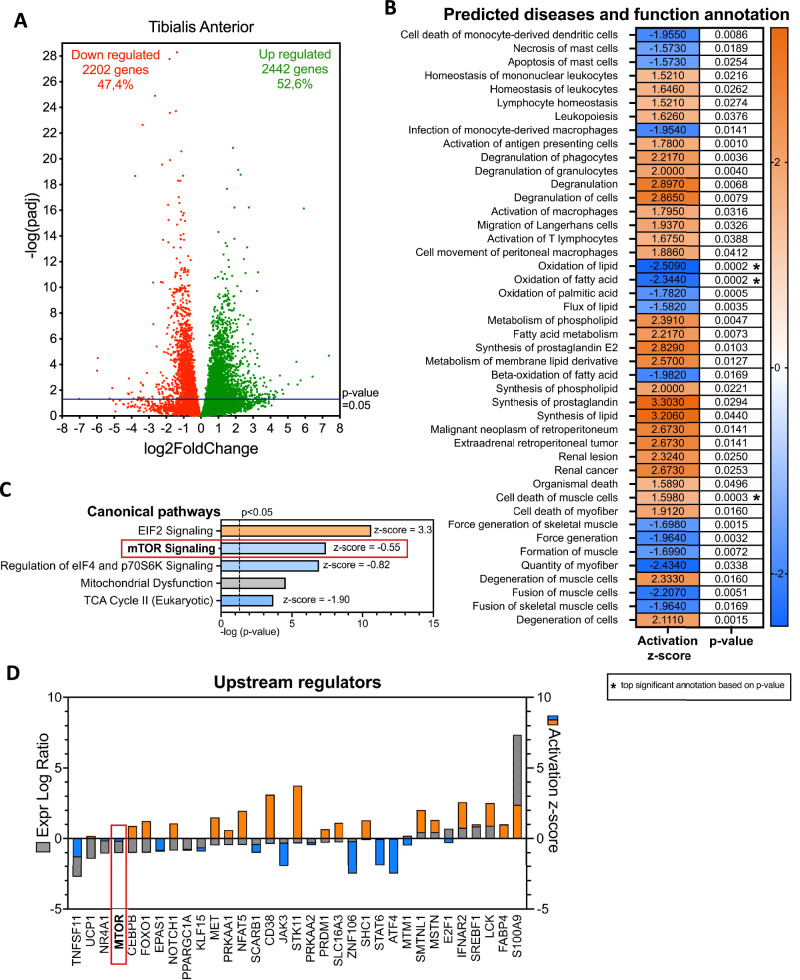
The mTOR signaling pathway is impaired in the skeletal muscle of Myf5-Cre-driven HuR knockout mice. **A** Volcano plot showing differential expression analysis (DESseq2) by log2fold changes following RNA-sequencing of mRNA isolated from the tibialis anterior muscle of Elavl1^fl/fl^ and Myf5-cre^/+^Elavl1^fl/fl^ mice. Analysis was done with *n* = 3 mice per group. Downregulated genes are indicated in red, and upregulated genes are indicated in green. The blue line indicates statistical significance at (*P* value = 0.05). Genes indicated in blue are known mRNA targets regulated by HuR. **B** Core analysis of top affected predicted diseases and function annotation from Ingenuity Pathway Analysis software (IPA: Ingenuity Systems®) following RNA-sequencing of the tibialis anterior muscle of Elavl1^fl/fl^ and Myf5-cre^/+^Elavl1^fl/fl^ mice. Table viewing annotation with z-scored of >1.5 or <−1.5. Asterix indicates the top three significant annotations based on the *P* value. Orange indicates an increase in value, and blue indicates a decrease in value. **C** Core analysis of top five canonical pathways from Ingenuity Pathway Analysis software (IPA: Ingenuity Systems®) following RNA-sequencing of the tibialis anterior muscle of Elavl1^fl/fl^ and Myf5-cre^/+^Elavl1^fl/fl^ mice. The dashed line indicates statistical significance at (*P* value = 0.05). Orange indicates an increase in value and blue indicates a decrease in value. Gray indicates no prediction of z-score assessment. Z-score is indicated on the right of each pathway. A red box indicates the pathway of interest. **D** Core analysis of upstream regulators from Ingenuity Pathway Analysis software (IPA: Ingenuity Systems®) following RNA-sequencing of the tibialis anterior muscle of Elavl1^fl/fl^ and Myf5-cre^/+^Elavl1^fl/fl^ mice. *X* axis shows upstream regulators. *Y* axis (left) shows the expression log ratio in gray. *Y* axis (right) shows the activation z-score, where orange indicates an increase in value and blue indicates a decrease in value. A red box indicates the upstream regulator of interest. See also Fig. S4.

The results above show that the Myf5-Cre-mediated depletion of HuR, unlike what was observed in MyoD-Cre-driven HuR knockout mice, results in the impaired formation of skeletal muscle and a reduction in exercise endurance. To establish mechanistically how this occurs, we performed RNA-sequencing experiments with total RNA isolated from the tibialis anterior muscle of both control and Myf5-Cre-driven HuR knockout mice. Volcano plot analysis of the acquired data shows that 4644 mRNAs were significantly affected (log2FC > 0.5 or < −0.5, *P* = 0.05) (Fig. 4A). Among these mRNAs, 47.4% were decreased while 52.6% were increased due to the genetic ablation of HuR. The validity and relevance of our RNA-Seq results shown in Fig. 4A were further supported by the observed predicted trend in the expression of previously established HuR mRNA targets, including MyoD, Myogenin, HMGB1, and nucleophosmin (Figs. 4A and S4). We next performed, using the Ingenuity Pathway Analysis software (IPA: Ingenuity Systems®), an analysis of disease-related pathways that may be altered by the depletion of HuR. We generated a list of predicted diseases affected by the Myf5-Cre-driven knockout of HuR in muscle by viewing a z-score of >1.5 or <−1.5 (Fig. 4B). We found, as expected, that many of the predicted diseases were related to defects in muscle function/formation (Fig. 4B). These results are consistent with our observation described above indicating that HuR is required for the formation and functionality of skeletal muscle.

**Fig. 5:**
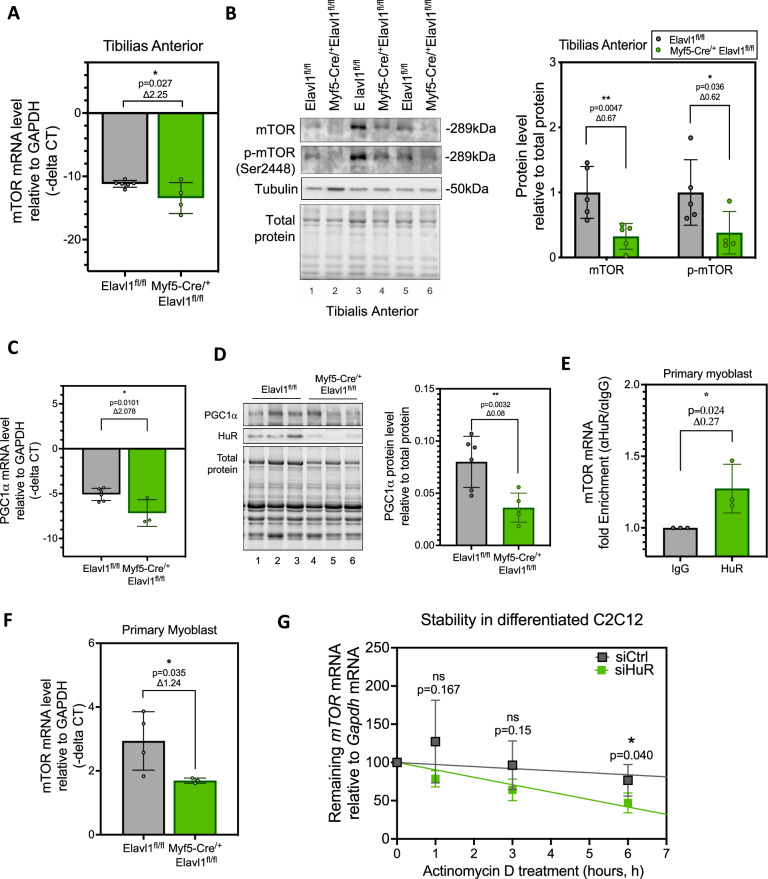
HuR stabilizes mTOR mRNA in muscle cells. **A** *mTOR* mRNA levels relative to GAPDH measured by RT-qPCR of mRNA from tibialis anterior muscle isolated from Elavl1^fl/fl^ and Myf5-cre^/+^Elavl1^fl/fl^ mice. **B** Left: Representative western blot analysis of mTOR protein, phosphorylation of mTOR at Ser2448, Tubulin, and Total protein in the muscle of Elavl1^fl/fl^ and Myf5-cre^/+^Elavl1^fl/fl^ mice. Right: Quantification of mTOR protein and phosphorylation of mTOR at Ser2448 normalized to Total protein. **C** *PGC1α* mRNA and **D** protein levels in tibialis anterior muscle of Elavl1^fl/fl^ and Myf5-Cre^/+^Elavl1^fl/fl^. **E** Fold enrichment of mTOR mRNA assessed by RT-qPCR following immunoprecipitation of HuR from primary myoblast isolated from the muscles of Elavl1^fl/fl^ mice. Results are normalized to the IgG negative control. **F** *mTOR* mRNA levels relative to *GAPDH* mRNA were measured by RT-qPCR from differentiated primary myoblasts isolated from the muscles of Elavl1^fl/fl^ and Myf5-cre^/+^Elavl1^fl/fl^ mice. **G** The stability of the *mTOR* mRNA was determined in C2C12 cells treated with a control (siCtl) or HuR-specific siRNA (siHuR) and treated with Actinomycin D (ActD) for 0, 1.5, 3, 4.5, or 6 h. mRNA was harvested, and levels of *mTOR* mRNA were then standardized against *GAPDH* mRNA levels and plotted relative to the level of mRNA at the 0 h ActD time point. The dot on the plot represents the mean with standard deviation. *n* = 3 per group. The statistics used were paired *t* tests (*P < 0.05). **A**–**D** Each point in the scatter dot plots represents an individual animal. The columns on the plots represent the mean with standard deviation. The statistics used were unpaired *t* tests (*P* < 0.05 = *, *P* < 0.01 = **). **E**–**G** Data are shown as the mean +/− standard deviation from *n* = 3 experiments. The statistics used were unpaired *t* tests (*P < 0.05).

To further decipher how the depletion of HuR is affecting these pathways, we next identified 5 canonical pathways that were significantly affected by the depletion of HuR (Fig. 4C). Many of these pathways, including the mTOR signaling pathway and the eIF4 and p70S6K pathways, are involved in regulating the general translation of mRNAs into proteins (Fig. 4C). One of the primary components found in these two first pathways is mTOR. mTOR, also known as the mechanistic target of rapamycin, is a serine-threonine kinase that regulates general translation and has been shown to mediate several cellular processes, including energy sensor, lipid metabolism, cellular growth, and autophagy [46]. Our data show thar mTOR, interestingly, was the only upstream regulator (from 33 upstream regulators) predicted to control multiple genes involved in the observed predicted diseases (with a *P* value of <0.05) that was also found in the top canonical pathways (Fig. 4D). Multiple studies have shown that the mTOR pathway plays a prominent role in regulating muscle formation and growth by affecting satellite cell activation, proliferation, and differentiation [47–50]. Our results suggest that HuR may contribute to the regulation of the myogenic program of satellite cells by regulating, in part, the expression of mTOR. Towards this end, we observed that *mTOR* mRNA levels were significantly decreased in muscle isolated from Myf5-Cre-driven HuR knockout mice when compared to their control counterparts (Fig. 5A). We also showed that both mTOR protein expression and mTOR activity (assessed by determining its phosphorylation on the Ser2448 residue) were reduced in the muscle of these mice (Figs. 5B and S5a).

One of the downstream targets of the mTOR pathway is PGC-1α (peroxisome proliferator-activated receptor gamma coactivator 1-alpha). We previously demonstrated that the enhanced oxidative capacity observed in MyoD-Cre driven HuR knockout mice results from increased stabilization of *PGC-1α* mRNA. Given that mTOR activity is diminished in Myf5-Cre-driven HuR knockout mice, we subsequently evaluated the expression levels of *PGC-1α* mRNA. We observed, in doing so, that the expression of *PGC-1α* mRNA and protein is significantly reduced in Myf5-Cre driven HuR knockout mice when compared to their wild-type control counterparts (Fig. 5C, D). These results thus further confirmed the effect of knocking out HuR on the activation of the mTOR pathway.

PAR-CLIP experiments have shown that HuR is capable of binding to *mTOR* mRNA in non-muscle cells [51, 52]. We thus performed RNP-immunoprecipitation (RIP) coupled to RT-qPCR experiments to assess if the *mTOR* mRNA is a target of HuR in muscle cells. We determined, using primary myoblast cultures and C2C12 murine myoblasts, that HuR indeed associates with this mRNA (Figs. 5E and S5b). Using both cell cultures, we observed, as we did in skeletal muscle, that the depletion of HuR decreases *mTOR* mRNA levels (Figs. 5F and S5c). We next assessed whether HuR was involved in regulating the stability of the *mTOR* mRNA. We, therefore, performed Actinomycin D pulse-chase experiments to determine the half-life of the *mTOR* mRNA in C2C12 cells depleted or not of HuR. We observed, under these conditions, a significant decrease in the stability of the *mTOR* mRNA in C2C12 cells depleted of HuR (Fig. 5G). Overall, our results indicate that the depletion of HuR in the Myf5+ cell population induced the inhibition of the mTOR signaling pathway due to a significant reduction in *mTOR* mRNA and protein levels.

### HuR mediated expression of mTOR results in the commitment of precursor cells towards the myogenic rather than the adipogenic lineage during muscle regeneration

**Fig. 6:**
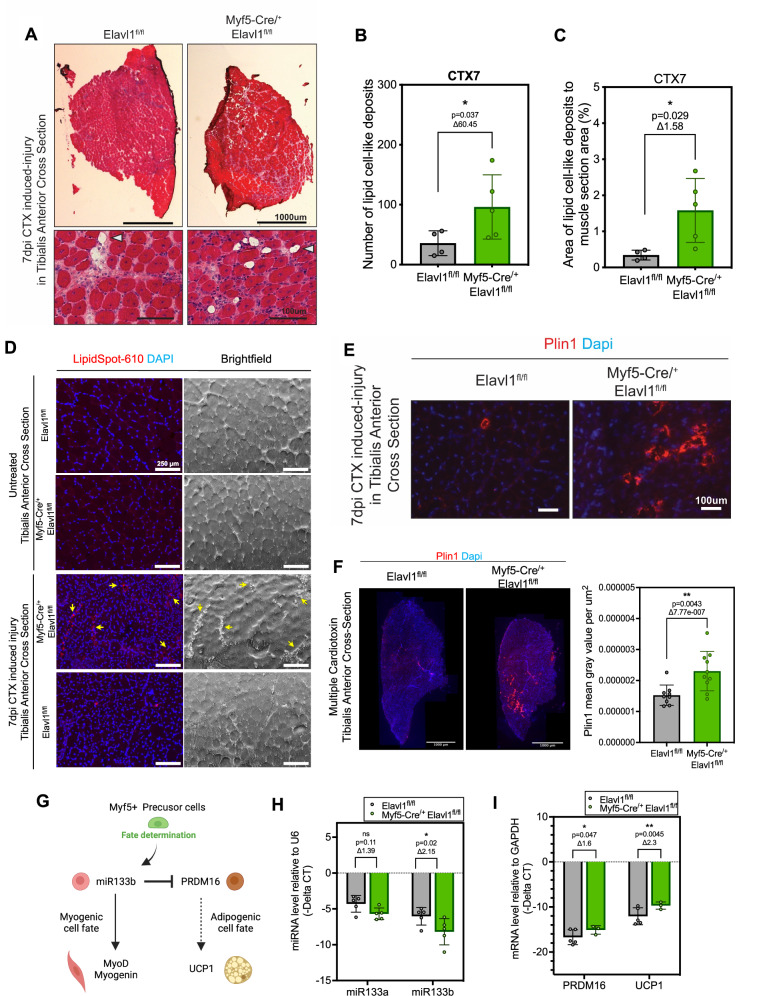
Depletion of HuR induces accumulation of lipid cell-like deposits following muscle regeneration. **A** Representative image of CTX-induced-injury 7 days post-injection in tibialis anterior muscle cross-sections stained by H&E. The top panels show a full cross-section of muscle, and the bottom panels show a zoomed image of the cross-section. Top Scale =100 μm and Bottom Scale =100 μm. The white arrow indicates lipid droplets. **B** The number of lipid cell-like deposits and **C** quantification of the total area covered by lipid cell-like deposits when normalized by muscle section area of CTX-induced-injury 7 days-post injection of tibialis anterior muscle cross-sections stained by H&E. **D** Representative immunofluorescence image of Lipid droplets using LipidSpot dye in tibialis anterior muscles obtained from untreated as well as CTX-injury (7 days-post injection) induced Elavl1^fl/fl^ control and Myf5-cre^/+^Elavl1^fl/fl^ mice. Left: LipidSpot, Right: Brightfield. Scale bar = 250 μm. **E** Representative immunofluorescence image of perilipin1 (plin1) staining on CTX-induced-injury 7 days-post injection of tibialis anterior muscle cross-sections of Elavl1^fl/fl^ and Myf5-cre^/+^Elavl1^fl/fl^ mice. Scale bar = 100 μm. **F** Representative immunofluorescence image of perilipin1 (plin1) staining on multiple CTX-induced injuries of tibialis anterior muscle cross-sections of Elavl1^fl/fl^ and Myf5-cre^/+^Elavl1^fl/fl^ mice. Scale bar = 1000 µm. Quantification of perilipin1 mean gray value per µm^2^. **G** Scheme of the molecular pathway leading to muscle precursor cell trans-differentiation into muscle cells or brown adipocytes. The scheme was generated using Biorender. **H** miR-133a and miR-133b levels relative to U6 measured by RT-qPCR of CTX-induced-injury 3 days post-injection of tibialis anterior muscle cross-sections of Elavl1^fl/fl^ and Myf5-cre^/+^Elavl1^fl/fl^ mice. **I***PRDM16* and *UCP1* mRNA levels relative to *GAPDH* mRNA measured by RT-qPCR of CTX-induced-injury 3 days post-injection of tibialis anterior muscle cross-sections of Elavl1^fl/fl^ and Myf5-cre^/+^Elavl1^fl/fl^ mice. **B**, **C**, **F**, **H**, **I** Each point in the scatter dot plots represents an individual animal. The columns on the plots represent the mean with standard deviation. The statistics used were unpaired *t* tests (<0.05 = *, <0.01 = **).

Several studies have shown that inhibition of the commitment of satellite cells during the early stages of muscle regeneration is associated with an increase in intramuscular fat [15, 53]. Under these conditions, the dysregulated commitment of these cells results in a change in their lipid biogenesis that, in turn, increases lipid content in muscle, leading to tissue impairment [54]. Interestingly, several studies have shown that altering the expression or function of mTOR in skeletal muscle increases ectopic adipogenesis as well as lipid droplet accumulation within this tissue [55] [56]. These effects, furthermore, were correlated with reduced muscle mass and a low endurance capacity. We therefore assessed whether the Myf5-Cre-mediated depletion of HuR, and the subsequent decrease in mTOR activation, has an effect on fat content in the muscle. We observed, in these mice, an increase in lipid cell-like deposits after muscle injury when compared to their wild-type control counterparts (Fig. 6A–C). We demonstrate, using a lipid droplet fluorescent stain, the increased formation of these lipid deposits in the muscles of knockout animals following injury (Figs. 6D and and S6). Interestingly, we did not observe any notable differences in lipid staining or the presence of lipid droplets between control and HuR-KO mice. These findings suggest that lipid accumulation in HuR-KO muscle is thus context-dependent and may be triggered or exacerbated by injury. By performing immunofluorescence experiments, we further show that these entities, which persist and accumulate following multiple injuries, are lipid droplets since they were positively stained for Perilipin1 (PLIN1), a lipid droplet-associated protein [57] (Fig. 6E, F). The increased presence of adipocytes in the muscle is further supported by our data showing that the depletion of HuR results in the decreased expression of the miR-133a, a pro-myogenic marker which inhibits the induction of the adipogenic program and the correlated significant increase in the expression of PRDM16 and the uncoupling protein 1 (UCP1), both of which are key markers of the adipogenic phenotype [7, 58] (Fig. 6G–I).

**Fig. 7:**
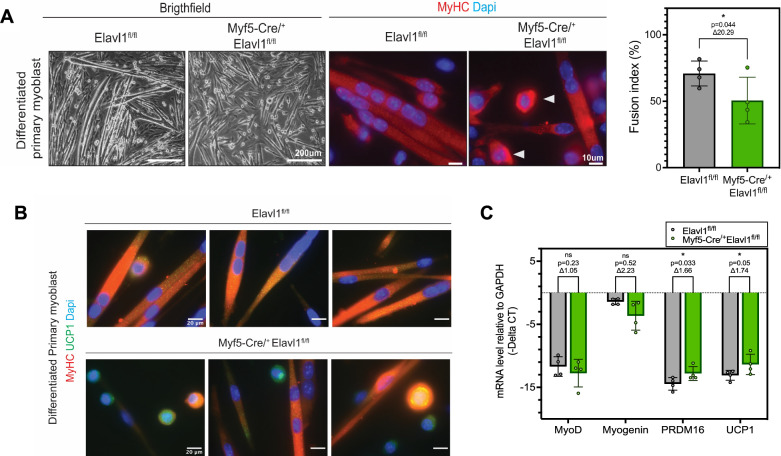
HuR prevents a BAT-like phenotype by supporting the initiation of the myogenic program in pre-committed muscle cells. **A** Differentiated primary myoblast from the muscles of Elavl1^fl/fl^ and Myf5-cre^/+^Elavl1^fl/fl^ mice and quantification of the fusion index. Cells of both groups were plated at 100% confluence before induction of differentiation. Left: Brightfield, Right: immunofluorescent images marked by MyHC and DAPI. Scale bar for brightfield images = 200 μm. Scale bar for immunofluorescent images = 10 μm. **B** Differentiated primary myoblast labeled with terminal markers of muscle MyHC and Brown adipose tissue UCP1 from the muscles of Elavl1^fl/fl^ and Myf5-cre^/+^Elavl1fl/fl mice. Cells of both groups were plated at 80% confluence before induction of differentiation. Scale bar = 20 μm. **C** Myogenic (*MyoD* and *Myogenin*) and adipogenic (*PRDM16* and *UCP1*) mRNA levels relative to *GAPDH* mRNA were measured by RT-qPCR from exponential primary myoblast isolated from the muscles of Elavl1^fl/fl^ and Myf5-cre^/+^Elavl1^fl/fl^ mice. **A**, **C** Each point in the scatter dot plots represents an individual animal. The columns on the plots represent the mean with standard deviation. The statistics used were unpaired *t* tests (<0.05 = *, <0.01 = **, *P* < 0.0001 = ****).

Several studies have shown that the unsuccessful commitment of muscle cells during the early stages of muscle regeneration is associated with an increase in intramuscular fat. We, therefore, investigated the potential link between the accumulation of intramuscular adipocytes during muscle regeneration in Myf5-Cre driven HuR knockout mice and the impaired commitment of muscle cells to the myogenic program. We, therefore, assessed the ability of primary myoblasts isolated from control as well as Myf5-Cre driven HuR knockout mice (which are marked by the decreased expression of mTOR) to differentiate into myotubes. We demonstrated that muscle cells isolated from the knockout mice do not differentiate into muscle fibers to the same extent as the control cells as shown by phase contrast images, as well as immunofluorescence experiments assessing the expression of MyHC (Fig. 7A). Although single myocytes are commonly observed in in vitro cultures, their increased abundance in the HuR-depleted group suggests a potential limitation in differentiation efficiency. To evaluate differentiation capacity, we therefore quantified the number of nuclei per myofibers. Indeed, the fusion capacity of Myf5-Cre^/+^Elavl1^fl/fl^-derived primary cells is significantly less than those isolated from their control counterparts (Figs. 7A and S7). These immunofluorescent experiments further showed that single myoblasts derived from primary cells isolated from knockout mice, unlike their control counterparts, adopt a round-like rather than a fusiform shape (Fig. 7A). We show, in these experiments, that a large percentage of these rounded cells were positive for UCP1 and that the proportion of positive UCP1 cells was higher in the Myf5-Cre^/+^Elavl1^fl/fl^ isolated primary cells than in their control counterparts (Fig. 7B). We observed, in isolated primary myoblasts from HuR knockout mice, a trend towards a decrease in the expression of mRNAs encoding MRFs such as MyoD and Myogenin and a significant increase in *PRDM16* and *UCP1* mRNA levels (Fig. 7C). These findings thus suggest that the depletion of HuR via Myf5-driven Cre, and the subsequent decrease in mTOR expression/activation, inhibits muscle cell myogenesis and instead promotes a modified differentiation program that, in part, leads to the development of brown adipocytes. Collectively, these results suggest a potential new function for HuR in the regulation of the Myo-adipose determination of muscle cells and that this function is dependent on the timing of HuR during the myogenic process and the HuR-mediated-posttranscriptional regulation of the *mTOR* mRNA.

## Discussion

**Fig. 8:**
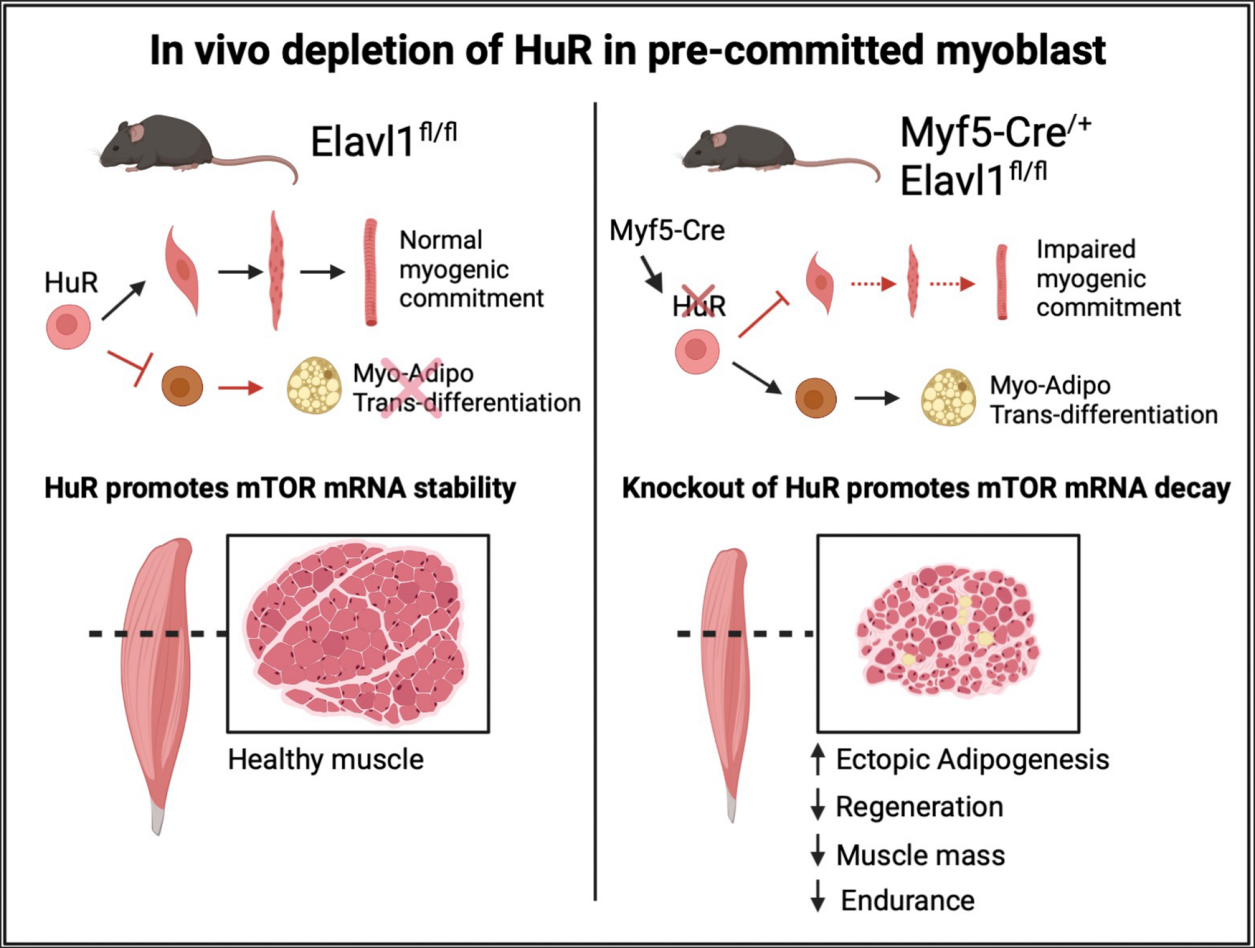
Model depicting the role of HuR in regulating the commitment of satellite cells to the myogenic program. Left: Under normal conditions, HuR regulates the commitment of satellite cells towards a myogenic program by regulating, in part, the stability of the mTOR mRNA. Right: Myf5-cre driven HuR knockout mice have defective myogenic commitment, due to a decrease in the stability of the mTOR mRNA, which reduces their differentiation into muscle. This, subsequently, results in an increase in the transition of these precursor cells towards an adipogenic program. The scheme was generated using Biorender.

In this work, we demonstrate that the genetic ablation of HuR during the phase of myogenesis where the fate of satellite cells is determined leads to a defect in muscle development, function, and regeneration. Using a Cre-LoxP strategy that involved the breeding of Myf5-Cre mice with Elavl1^fl/fl^ mice [59] we show that our HuR knockout mice (Myf5-Cre^/+^Elavl1^fl/fl^) are characterized by a reduction of muscle mass (when compared to the control counterparts) which is due to a decrease in fiber number and size. We demonstrate that muscle precursor cells, in these mice, have defective myogenic commitment which reduces their differentiation into muscle. Our data suggest that this effect is due, in part, to the decreased activation of the mTOR signaling pathway caused by the decreased stability of the mTOR mRNA that, in turn, significantly reduced its protein expression (Fig. 8). In addition, this lack of myogenic commitment, which is likely due, in part, to the decreased expression/activation of mTOR, was accompanied by an increase in the transition of these precursor cells towards an adipogenic program which affected the global phenotypic profile of the muscles.

We have previously shown that HuR plays an important role, *in cellulo*, using the C2C12 cell culture model, in the differentiation of myoblasts into myotubes. However, considering the complexity of muscle structure and HuR function, it is important to understand how HuR functions in promoting the myogenic process in vivo [11, 19, 31–33, 36, 37, 39, 40]. Our previously published results showed that depleting HuR during the later stage of myogenesis (due to the expression of Cre using a MyoD promoter) does not affect muscle mass and integrity. Our results showed that it rather affects the composition of muscle fibers, leading to an alteration in their metabolic phenotype that increases their endurance capacity [31]. In this present study, however, we show that depleting HuR at an earlier stage of the myogenic process (using Myf5-Cre) affects both the formation and function of skeletal muscle, resulting, respectively, in a decrease in its mass and endurance capacity. The differences observed between the two models are likely due to the differential temporal expression of Myf5 and MyoD during embryogenesis as well as during muscle regeneration. Indeed, while the expression of Myf5 in satellite cells is known to determine the commitment of muscle precursor cells towards the myogenic lineage, MyoD expression occurs once these cells proliferate into myoblasts. The fact that depleting HuR during the pre-commitment (Myf5-Cre) vs. post-commitment (MyoD-Cre) phase has two opposite phenotypes on the outcome of muscle development and function highlights the complex role of HuR in muscle homeostasis. Although our results demonstrate that the function of HuR in skeletal muscles is dictated by the stage of the myogenic process, what remains unknown, however, is the mechanisms (i.e., trans-acting factors, posttranslational modifications) that differentially dictate these phenotypic outcomes.

Here, we provide the first proof that HuR is required in precursor muscle cells for myogenesis in vivo during the early stages of the myogenic process. Our work uncovers a novel function for HuR during this process, where HuR executes its pro-myogenic role both during embryogenesis and upon injury to muscle. We suggest that the Myf5-Cre-mediated depletion of HuR from precursor muscle cells affects the formation of skeletal muscle and, furthermore, prevents their regeneration upon injury (likely due to the increased formation of lipid deposits), thus impairing their endurance capacity. Several studies have shown the importance of lipid content in regulating muscle stem cell fate during the myogenic program [54]. Additionally, in vivo models of lipid overload were shown to induce a decrease in muscle mass and endurance independently of the fiber-type specification [60]. These observations are consistent with the observed phenotype in our HuR knockout mice (Myf5-Cre^/+^Elavl1^fl/fl^). HuR has been suggested to be a negative regulator of adipogenesis due to its ability to modulate the expression of the Adipose triglyceride lipase (ATGL) and Insulin-induced gene 1 (insig-1) in adipose tissues [61, 62]. However, these studies did not determine the mechanisms through which HuR modulates adipogenic content in muscle cells.

Our work addresses this gap and identifies mTOR mRNA as a novel HuR target in muscle precursor cells. By stabilizing the mTOR mRNA to promote the expression of its protein, HuR ensures that these muscle cells commit to the myogenic process. Additionally, our data also suggest that by promoting mTOR expression, HuR prevents the drift of muscle progenitor cells toward the adipogenic process. Interestingly, the phenotype we report here, with the Myf5-Cre driven muscle-specific HuR KO, is consistent with what was observed in mTOR knockout mice as well as mice where mTOR kinase activity was inhibited [47, 48, 55, 56]. Indeed, muscle-specific mTOR knockout mice have decreased overall body weight associated with a decrease in myofiber cross-sectional area. These mice also displayed a decrease in oxidative metabolism and showed lipid droplet accumulation within muscles [56]. Furthermore, the inactivation of mTOR kinase activity resulted in a reduced muscle mass that was associated with an increase in ectopic adipogenesis within the muscle tissue leading to low endurance capacity [55]. The phenotypes observed in our Myf5-Cre^/+^Elavl1^fl/fl^ mice model, therefore, which is characterized by a decrease in muscle mass and endurance, likely occur due to the impaired commitment of muscle precursor cells because of the decreased stability of the mTOR mRNA.

Interestingly, mTOR genetic ablation or modification has been shown to impair development. Indeed, embryos stop developing properly during embryogenesis around E8.5 and die at E12.5 [63–65]. This is comparable to the importance of HuR during embryogenesis, where conventional depletion of HuR leads to stage retardation at E8.5, which induces lethality from E10.5 to E12.5 [59]. However, whether HuR regulates mTOR in early embryogenesis remains unknown. Although HuR was found to associate with mTOR mRNA in HEK293 cells [51, 66] and, as described in this study, in muscle cells, whether HuR regulates mTOR mRNA expression in other cell types remains to be discovered.

The critical role of HuR and mTOR in development, particularly in muscle formation, endorses the validity of pursuing further studies involving the HuR-mTOR mRNA interactions. Here we show, for the first time, the relation between HuR and the post-transcriptional regulation of the mTOR mRNA. mTOR is a component of two different complexes (mTORC1 and mTORC2) that play distinctive roles in cells. Of the two, mTORC1 is known to be rapamycin-sensitive and is the principal complex involved in regulating PGC-1α, particularly in skeletal muscle, where it enhances mitochondrial biogenesis and supports oxidative metabolism. Our data showing that the expression of PGC-1α mRNA and protein levels are decreased in the muscle of the knockout mice suggests that the mTORC1 complex is specifically targeted under these conditions.

Thus, in considering the important function of mTOR in multiple cellular processes, underlining how HuR is involved in regulating the stability of the mTOR mRNA may help establish interesting therapeutic avenues that can be used to prevent the onset of diseases associated with dysfunctions in these processes.

## Materials and methods

### Approvals of protocols/experimental procedures

All experimental protocols and methods were approved by the McGill Biological Safety Committee in accordance with guidelines and regulations mandated by the Canadian Biosafety Standards (CBS) of the Public Health Agency of Canada (PHAC).

### Animals and breeding strategies

All in vivo models and experiments were approved by the McGill University Faculty of Medicine, Animal Care Committee, and followed the Canadian Council of Animal Care guidelines. In addition, all experiments with animals were performed in accordance with relevant guidelines and regulations. Mice were housed in a controlled environment with a 12 h/12 h light cycle and a room temperature of 22 °C. MyoD-Cre/+; Elavl1^fl/fl^ were generated as previously described [31]. Myf5-Cre/+Elavl1^fl/fl^ model was generated following the same strategy using a non-pathogenic mouse on a C57BL/6 background expressing Cre recombinase under the control of the Myf5 promoter (Myf5-Cre/+) [67] to delete exon 2 of the Elavl1 gene floxed by loxP sites (Elavl1^fl/fl^) [59]. The muscle-specific knockout mice were maintained by breeding Myf5-Cre/+; Elavl1^fl/fl^ mice with Elav1^fl/fl^ mice and using Cre recombinase negative mouse (Myf5Cre-/- Elavl1^fl/fl^) as control littermates. All in vivo experiments were done using 2-month-old males. Body measurements were done on mice by Bucco-Anal measurement of body length in centimeters, weight in grams, and body ratio representing body weight in grams divided by body length in centimeters. Harvested tissues were weighed and normalized to the body length of the animal. Experiments focus mainly on the Tibialis Anterior muscle unless stated otherwise. Other tissues, such as the soleus (Sol.), gastrocnemius (Gast.), extensor digitorum longus (EDL), peroneus (Per.), heart, and white adipose tissue, were analyzed for knockout validation and weight. Strain references are presented in *key resource tables*.

### Genotyping

Genotyping was performed using genomic DNA isolated from tail cuts (stored at −20 °C until genotyping) or muscle tissue samples (stored at −80 °C until genotyping) as described in [31]. Primer sequences are presented in key resource tables.

### Tissue processing, sectioning, staining, and analysis

Samples were either fast-frozen in liquid nitrogen until processing for protein and RNA or mounted on 7% tragacanth gum, followed by isopentenyl freezing and stored at −80 °C before processing or cryo-sectioning. Muscles were sectioned at 10 mm thickness at −25 °C using a cryostat (HM 525-Thermo). Sections used for analysis correspond to the largest area in the periphery of the muscle, which was reached by measuring 2 mm from the top of the tibialis anterior muscle. Muscle sections were stained with hematoxylin and eosin (H&E) or were used for immunofluorescent experiments using antibodies specific for Perilipin A/B (Sigma #P1873, 1:250), laminin (Sigma #L9393, 1:500), eMyHC (DSHB #F1.652-b, 1:100), Pax7 (DHSB 1:100), or using the immunofluorescent dye Lipidspot-610. Satellite cell basal number was done manually using a cell counter from ImageJ FIJI on immunofluorescent images, and the investigator was blinded to the group during satellite cell basal number counts. The surface area and the number of lipid droplets were determined manually using ImageJ FIJI on H&E images. Cross-sectional area analysis was done manually using ImageJ FIJI for H&E images or semi-automatically by Open-CSAM for immunofluorescence staining with Laminin (Sigma #L9393, 1:500), followed by manual correction. The protocol for Open-CSAM was followed as described in [68]. Softwares used are presented in key resource tables.

### Fiber-type classifications

Muscle was sectioned and assessed for fiber-type classification by immunofluorescence as described in [69]. Sections were air-dried for 10 min and then blocked with 10% goat serum (Gibco #16210-064). No fixing or permeabilization was used. Sections were surrounded by a hydrophobic pen and incubated overnight at 4 °C with a cocktail mix of primary antibodies (type IIa – SC-71; Developmental Studies Hybridoma Bank, 1:600, IIb – BF-F3; Developmental Studies Hybridoma Bank, 1:100, and Laminin; Sigma #L9393, 1:500). Sections were washed 3 times with PBS and incubated for 1 h at room temperature with a cocktail mix of secondary antibodies (Alexa Fluor 488 IgG_1_, Invitrogen, 1:500, Alexa Fluor 555 IgM_,_ Invitrogen, 1:500, Alexa Fluor 647 IgG_,_ Invitrogen, 1:500). Unstained fibers were classified as Type IIx. Sections were washed 3 times with PBS and then mounted with *Vectasheild* HardSet Antifade Mounting Medium with DAPI (Vector Laboratory #H-1500). Pictures were taken within 24 h following the immunofluorescence protocol. A Zeiss confocal microscope was used to take a sequential picture of the full section. Each filter was mounted into full-section images of 16-bit grayscale by the mosaic macro of ImageJ FIJI. Laminin images were modified by sharpening and finding edges to accentuate the delimitation of fibers. Protocol for MuscleJ was followed as described in [70]. Softwares used are presented in key resource tables.

### Grips test

The protocol for the grip test was followed as described in [31]. The grip Test apparatus used was BIOSEB’s Grip Strength Test (Model GT3) and associated BIOSEB software (BIO-CIS- Version 1.5.1.0) for data collection. Softwares used are presented in key resource tables.

### Exhaustion test

The protocol for the exhaustion test was followed as described in [31] with modification using 0.5 mA for the electric grid. Exhaustion was considered when the mouse was 5 s on the grid, which automatically stops with the conditional parameters or at the experiment endpoint of 80 min running on a treadmill. The treadmill used was the Five Lanes Touchscreen Convertible Treadmill for mice (Panlab, Model LE8710MTS) from Harvard Apparatus and data was collected using SEDACOM software V2.0. Softwares used are presented in key resource tables.

### Cardiotoxin injury

Muscle CTX-induced injury experiments were as described in [43]. Mice were anesthetized using isoflurane before intramuscular injection of cardiotoxin. Three intramuscular injections of 10 μM Cardiotoxin (Cedarlane # L8102) using a Hamilton syringe were done in the tibialis anterior muscle of the mouse. Mice were given carprofen for analgesia and monitored for 24 h after injection. Muscles were collected after 3, 7, 10, 14, or 21 days of regeneration. For the multiple injury experiment, 3 injections were done following the same protocol with 21 days between each injection. Harvest was done 21 days after the last injection (a total of 63 days following the first injection).

### Primary myoblast and cell culture

Satellite cells were isolated from the diaphragm and abdominal skeletal muscle of 8–12-week-old mice using. The mouse Satellite Cell Isolation protocol was adapted from the MACS isolation kit protocol by Miltenyi Biotec. Trypsin (ThermoFisher) and Collagenase D (Sigma) were used for tissue digestion, followed by column filtration Steriflip (Millipore) and red blood cell removal by Red Blood Cell Lysis buffer (R7757-100ml, Sigma). Isolation of muscle satellite cells was done using the Satellite cell isolation kit mouse (Miltenyi Biotec) and Anti-Integrin a7 beads (Miltenyi Biotec) combined to LS Columns (Miltenyi Biotec) placed on QuadroMACS™ Separator (Miltenyi Biotec) and Macs multi stand (Miltenyi Biotec). Approximately 60,000 to 80,000 cells were isolated per mouse. Cells were isolated and plated for either satellite cell analysis on the day of isolation or for differentiation. For satellite cell analysis, 50,000 cells resuspended in 100 μl of Sc media were plated on a 2-cm^2^ surface coated with 500 μl of 0.2% gelatin in one well of a six-well plate. For differentiation, 50,000 cells were resuspended in 100 μl of Sc media and plated on a 2-cm^2^ surface coated with 500 μl of 0.2% gelatin in one well of six-well plates. Media was changed the next day with fresh satellite cell media (DMEM glutamax; ThermoFisher, F12-Glutamax; ThermoFisher, 10% FBS (Sigma), 1% Ult. G (Cederlane) and changed to differentiating media (DMEM, 2% Horse Serum, ThermoFisher) when cells reached 90–100% confluency, which is equivalent to Day 0 of differentiation (DM 0). Immunofluorescent experiments were done on primary cells using antibodies binding to MyHC (MF-20, Developmental Studies Hybridoma Bank, 1:250), or UCP1 (Abcam), followed by manual analysis using a cell counter from ImageJ FIJI. The fusion index was measured by calculating the ratio of the average number of nuclei per myotube divided by the total number of nuclei within myotubes in the same field. C2C12 murine myoblast (ATCC, Manassas, VA, USA) were cultured in growth media (20% Fetal Bovine Serum (FBS) and 1% penicillin/streptomycin antibiotics in Dulbecco’s modified Eagle medium (DMEM) (Invitrogen). Cells were grown in a humidified incubator at 37 °C, 5% CO_2_.

### RNA-immunoprecipitation experiment

RNA-Immunoprecipitation experiments were done on either C2C12 or primary myoblasts. Protein A beads were pre-washed with low salt buffer (50 mM Tris, pH 8, 0.5% Triton X-100, 150 mM NaCl, 1× protease inhibitors) and incubated for 4 h at 4 °C with antibodies against IgG or HuR (3A2). Beads were then incubated overnight at 4 °C with 800 mg of total cell extracts from either C2C12 or primary myoblast followed by the extraction of the associated immunoprecipitated RNA, which was resuspended in nuclease-free water. The RNA was subsequently used for RT-qPCR experiments as described below.

### Treatment with siRNAs

Transfection of siRNA specific for HuR and control siRNA was done in C2C12 as previously described using jetPRIME (Polyplus transfection reagent) according to the manufacturer’s instructions [19]. The siRNA sequences are presented in *key resource tables*.

### Actinomycin D pulse-chase

An Actinomycin D pulse-chase experiment (ActD) was done using C2C12 cells transfected with scrambled control or siRNAs against HuR. Cells were then treated for 0 h, 1 h, 3 h, and 6 h with 2.5 μg/ml of the RNA polymerase II inhibitor, actinomycin D (Act. D) (Sigma, A1410) to assess the stability of mRNA. mRNA levels were plotted relative to mRNA levels (considered 100%) at 0 h of Act. D treatment.

### mRNA/miRNA isolation and reverse transcription-quantitative PCR (RT-qPCR)

Total RNA was isolated from muscle tissue by adding 1 ml of TRIzol reagent (Ambion) per 50–100 mg of tissue. The tissue was then mechanically homogenized. C2C12, primary, and satellite cells were processed by adding 50 μl of Trizol per cell pellet. mRNA was quantified using the NanoDrop UV spectrophotometer (Thermo Fisher Scientific Inc). Reverse Transcription was done with 1 μg of the sample using iScript™ Reverse Transcription Supermix (Bio-Rad#170884). The dilution of cDNA used for quantification was 1/20 for satellite cell samples and 1/40 or 1/80 for tissues, C2C12, and primary myoblasts, respectively. Quantitative PCR was performed using Sso Fast EvaGreen ® Supermix (Bio-Rad# 1725204) and a Corbett RG-6000. Expression levels were standardized to the reference gene GAPDH. Expression levels were measured by using the delta CT (cycle threshold) of the target gene and reference gene (GAPDH). Negative delta CT was used to plot the figures. Primer sequences are presented in *key resource tables*. For miRNA analysis, miRCURY LNA miRNA PCR assay (Qiagen #3621594) was used with target miRNA UniSp6 (yp00203954), hsa-miR-133b (yp00206058), and miRNA hsa-miR-133a-3p (yp00204788) from Qiagen.

### Protein isolation and immunoblotting

Protein extraction from frozen muscle tissues was performed using Muscle Protein Extraction Buffer (MPEB)(1× PBS,1% NP-40, 0.5% DOC, 0.1% SDS, 2 mM SOV, 1× protease inhibitor (Roche)) followed by mechanical homogenization and 1 h rotation at 4 °C. C2C12 or isolated primary myoblast were lysed in RIPA buffer by 3 cycles of 30 s vortex- 5 min incubation on ice. The supernatant was collected after centrifugation at 12,000 rpm for 15 min at 4 °C and quantified by Bradford. Western blot was performed using a Trans-Blot turbo, Transfer System (Bio-Rad). Total protein was used for normalization and was measured using tris-glycine extended (TGX) gel. Membranes were blocked with 5% milk in TBST or EveryBlot Blocking Buffer (Bio-Rad), then probed for HuR (3A2, 1:5000), Tubulin (1:1000), mTOR (Cell signalling, 1:5000), Phospho-mTOR (Ser2448) (Cell Signalling, 1:2000), OXPHOS cocktail (Abcam, 1:5000). OXPHOS complex II (SDHA) (Cell Signaling mAb #11998, 1:1000), α-Tubulin (Developmental studies Hybridoma Bank, 1:1000), Myoglobin (abcam #11998, 1:1000), PGC-1α (abcam, 1:1000). Antibodies used are presented in key resource tables.

### mRNA sequencing (RNA-seq) and Ingenuity pathway analysis (IPA)

Total RNA extracted from the tibialis anterior muscle was used for mRNA sequencing experiments. RNA-seq libraries were sequenced on the Illumina NextSeq 500 platform by the Institute for Research in Immunology and Cancer (IRIC) Genomics Core Facility, University of Montreal. Differential expression was done using the DESeq2 package between Myf5-Cre^/+^Elavl1^fl/fl^ and control Elavl1^fl/fl^. Ingenuity pathway analysis Software 5.0 (IPA, Ingenuity Systems) was then used to analyze the RNA-seq dataset generated by DESeq analysis to identify altered biological pathways. Parameters to narrow selected pathways were based on *P* value or z-score. Reference to affiliated dataset presented in key resource tables (to be deposited shortly to genebank).

### Statistical analysis and reproducibility

GraphPad Prism (Version 9.3.1) was used for statistical analyses. The significance of the difference in experiments comparing the control group to the knockout group *was assessed by an unpaired* t *test. F test* was used to compare variances was used to confirm similar variance between groups that are statistically compared, if the variation was significant, a Welch’s *t* test was applied. All values are plotted column bar graph representing the mean with a standard deviation, and each scattered point represents an individual animal. The statistics used for the treadmill population experiment were a Log-rank (Mantel–Cox) test. Significance, *P* values (p), and effect sizes (Δ) are displayed on all graphs. The *P* *values* higher than 0.05 represent non-significant (ns) values. *P* values equal to or less than 0.05 represent significant values (<0.05 = *, <0.01 = **, <0.001 = ***, <0.0001 = ****). In vitro experiments were reproduced three times, and in vivo experiments used an average of 8 mice (*n* = 4–12).

#### Ethics statement

All animal experiments were approved by the McGill University Faculty of Medicine Animal Care Committee and performed in accordance with the Canadian Council on Animal Care guidelines and institutional regulations. This study did not involve human participants or human-derived data.

## Supplementary information

Supp Material

## Data Availability

Any additional information required to reanalyze the data reported in this paper is available from the corresponding contact upon request.
