# Supplementary material for: HuR-mediated regulation of mTOR mRNA stability promotes the commitment of satellite cells towards myogenesis
Source: Cell Death Dis. 2026 Apr 21;17(1):527. doi: 10.1038/s41419-026-08759-1 (PMC13230890; doi:10.1038/s41419-026-08759-1)
Supplement: Supplementary file 1 — Supp Material [file 41419_2026_8759_MOESM1_ESM.pdf]

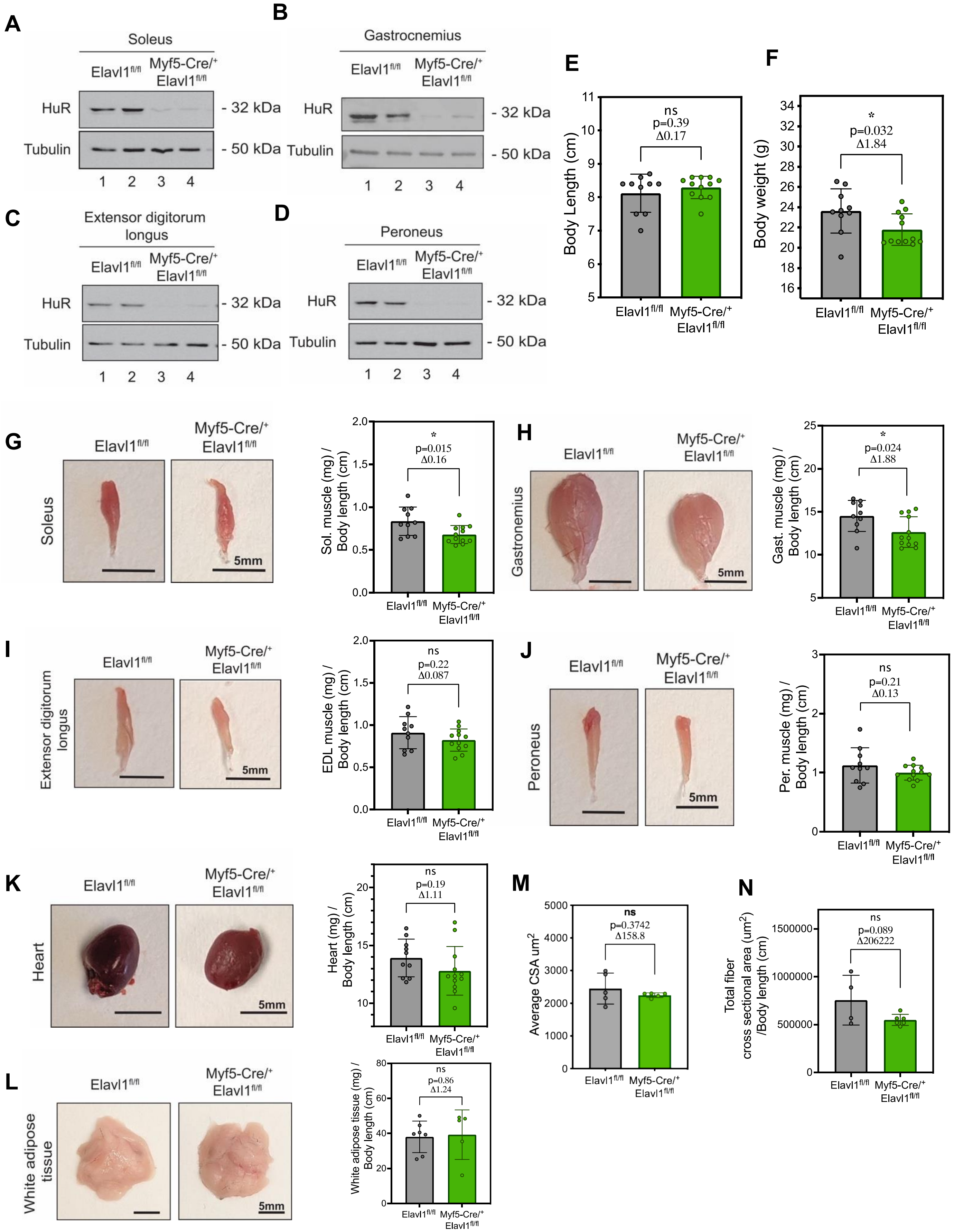

A
Soleus
Elavl1fl/fl
Myf5-Cre/+ Elavl1fl/fl
HuR
Tubulin
- 32 kDa
- 50 kDa
1 2 3 4
B
Gastrocnemius
C
Extensor digitorum longus
D
Peroneus
E
Body Length (cm)
ns
p=0.39
Δ0.17
F
Body weight (g)
*
p=0.032
Δ1.84
G
Soleus
5mm
Sol. muscle (mg) / Body length (cm)
*
p=0.015
Δ0.16
H
Gastronemius
Gast. muscle (mg) / Body length (cm)
*
p=0.024
Δ1.88
I
Extensor digitorum longus
EDL muscle (mg) / Body length (cm)
ns
p=0.22
Δ0.087
J
Peroneus
Per. muscle (mg) / Body length (cm)
ns
p=0.21
Δ0.13
K
Heart
Heart (mg) / Body length (cm)
ns
p=0.19
Δ1.11
L
White adipose tissue
White adipose tissue (mg) / Body length (cm)
ns
p=0.86
Δ1.24
M
Average CSA um2
ns
p=0.3742
Δ158.8
N
Total fiber cross sectional area (um2) /Body length (cm)
ns
p=0.089
Δ206222

Fig. S1. Validation of skeletal muscles depleted of HuR mouse model (Myf5-cre$^{/+}$Elavl1$^{fl/fl}$), related to Figure 1.
(**A-D**) Validation, by western blot, of HuR knockout in **(A)** soleus, **(B)** gastrocnemius, **(C)** extensor digitorum longus and **(D)** peroneus skeletal muscles of Elavl1$^{fl/fl}$ and Myf5-cre$^{/+}$Elavl1$^{fl/fl}$ mice. n=2 animals. **(E)** Bucco-Anal measurement of body length in centimetres of Elavl1$^{fl/fl}$ and Myf5-cre$^{/+}$Elavl1$^{fl/fl}$ mice. **(F)** Bodyweight in grams. **(E, F)** Each point in the scatter dot plots represents an individual animal. The columns on the plots represent the mean with standard deviation, and unpaired t-test (*=p<0.05). (**G-J**) **Left:** Representative picture of the soleus (Sol.) **(G)**, gastrocnemius (Gast.) **(H)**, extensor digitorum longus (EDL) **(I)** and peroneus (Per.) **(J)** skeletal muscles of Elavl1$^{fl/fl}$ and Myf5-cre$^{/+}$Elavl1$^{fl/fl}$ mice. **Right:** Quantification of skeletal muscle mass shown on the left, normalized to body length on the right panel. Scale bar = 5mm. **(G-J)** Each point in the scatter dot plots represents an individual animal. The columns on the plots represent the mean with standard deviation and are analyzed using an unpaired t-test (*=p<0.05). (**K-L**) **Left:** Representative picture of a heart **(K)** or white adipose tissue **(L)** of Elavl1$^{fl/fl}$ and Myf5-cre$^{/+}$Elavl1$^{fl/fl}$ mice. **Right:** Quantification of tissue mass normalized to body length. Scale bar = 5mm. **(K-L)** Each point in the scatter dot plots represents an individual animal. The columns on the plots represent the mean with standard deviation. The statistics used were unpaired t-tests. **(M)** Average cross-sectional area (mm^2) of tibialis anterior skeletal muscle. **(N)** Quantification of total cross-sectional area (mm^2) of tibialis anterior skeletal muscle normalized to body length of Elavl1$^{fl/fl}$ and Myf5-cre$^{/+}$Elavl1$^{fl/fl}$ mice. Each point in the scatter dot plot represents an individual animal. The columns on the plot represent the mean with standard deviation. The statistics used were unpaired t-tests.

.

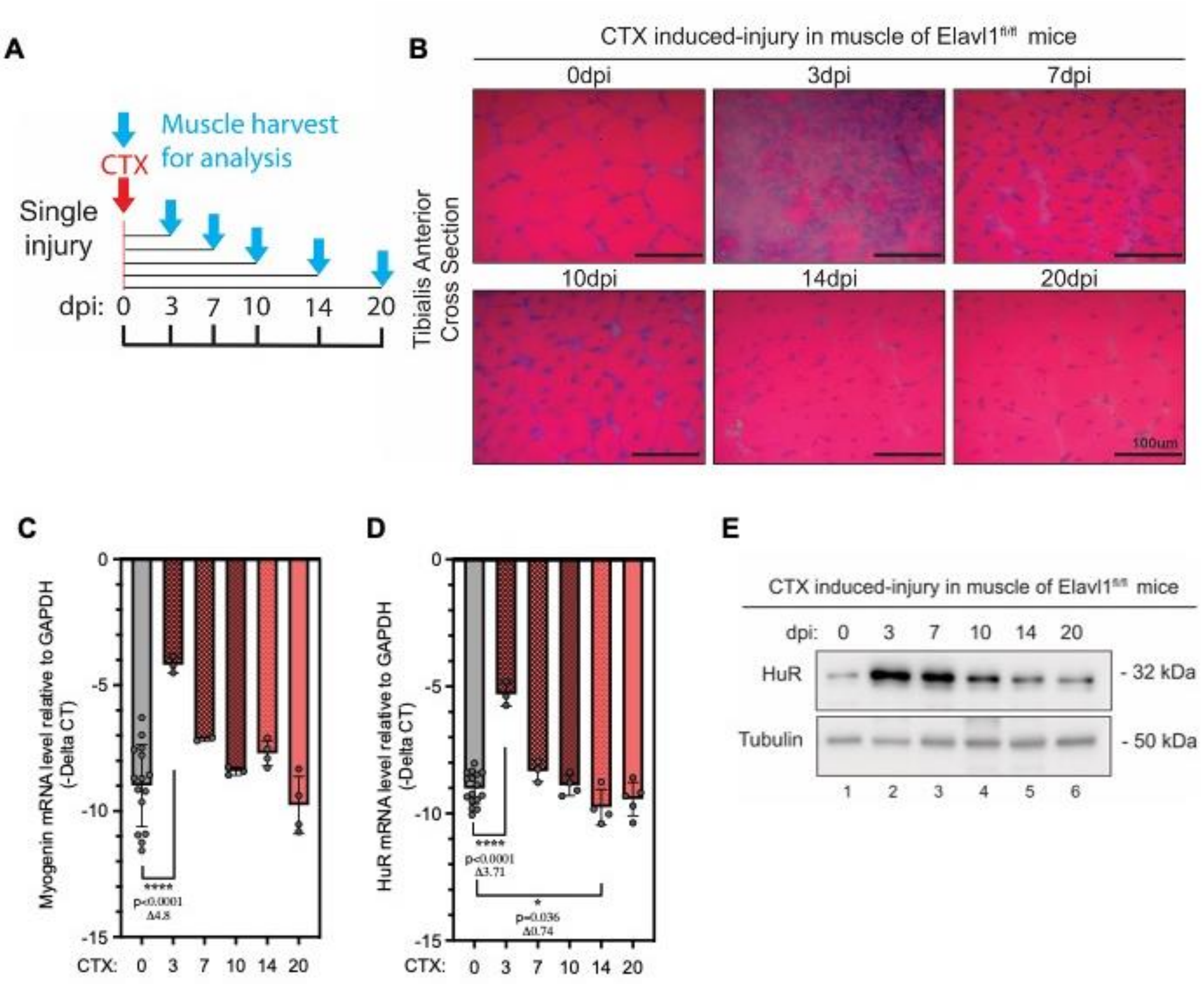

Fig. S2. HuR expression increases during the early stages of cardiotoxin-induced muscle regeneration, related to Figure 3.

(A) Scheme of cardiotoxin-induced muscle regeneration experiment in control Elavl1$^{fl/fl}$ mice. **(B)** Cross-section of tibialis anterior muscle stained with H&E after cardiotoxin-induced muscle regeneration. **(C)** *Myogenin* mRNA level relative to *GAPDH* mRNA measured by RT-qPCR in tibialis anterior muscle after cardiotoxin-induced muscle regeneration. Scale bar = 100μm. **(D)** *HuR* mRNA level relative to *GAPDH* mRNA measured by RT-qPCR of tibialis anterior muscle lysis after cardiotoxin-induced muscle regeneration. **(E)** HuR protein levels were analyzed by western blot after cardiotoxin-induced muscle regeneration, Tubulin protein levels are shown as a loading control. (**C, D)** Each point in the scatter dot plots represents an individual animal. The columns on the plots represent the mean with standard deviation. Unpaired t-tests between the untreated group and each time point of the cardiotoxin-induced muscle regeneration treated groups (p<0.05=*, p<0.0001=****).

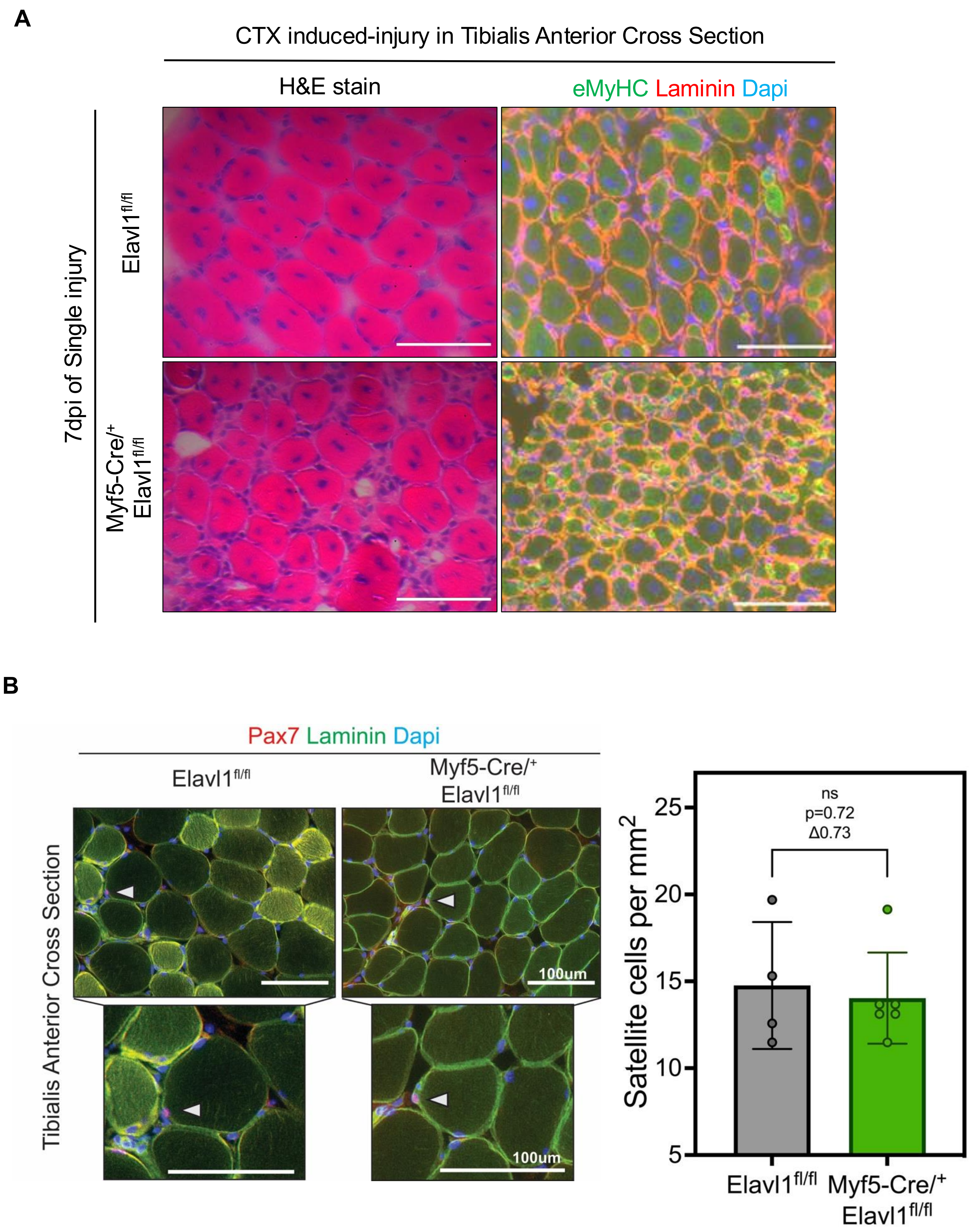
A
CTX induced-injury in Tibialis Anterior Cross Section
H&E stain
eMyHC Laminin Dapi
7dpi of Single injury
Elavl1fl/fl
Myf5-Cre/+
Elavl1fl/fl
B
Pax7 Laminin Dapi
Elavl1fl/fl
Myf5-Cre/+
Elavl1fl/fl
Tibialis Anterior Cross Section
100um
100um
Satellite cells per mm2
ns
p=0.72
Δ0.73
25
20
15
10
5
Elavl1fl/fl
Myf5-Cre/+
Elavl1fl/fl

Fig. S3. The Myf5-Cre driven knockout of HuR induces defective muscle regeneration without affecting the basal number of satellite cells, related to Figure 3. (A) Zoomed in cross-sections of injured tibialis anterior muscles at 7dpi shown in Figure 3B **Left:** stained with H&E. **Right:** labelled for newly formed fibers as assessed by immunofluorescence of embryonic myosin heavy chain (eMyHC, green). Staining for Laminin (red) was used for fiber perimeter and for nuclei by DAPI (blue). Scale bar = 100mm. **(B)** Immunofluorescence image of satellite cells stained for Pax7 (red) in the untreated tibialis anterior muscle of Elavl1$^{fl/fl}$ and Myf5-cre$^{/+}$Elavl1$^{fl/fl}$ mice. Fiber perimeter stained by laminin (green). The white arrow indicates Pax7+ satellite cells. Scale =100mm. Quantification of the basal number of satellite cells normalized by mm^2. Each point in the scatter dot plots represents an individual animal. The columns on the plots represent the mean with standard deviation. The statistics used were unpaired t-tests.

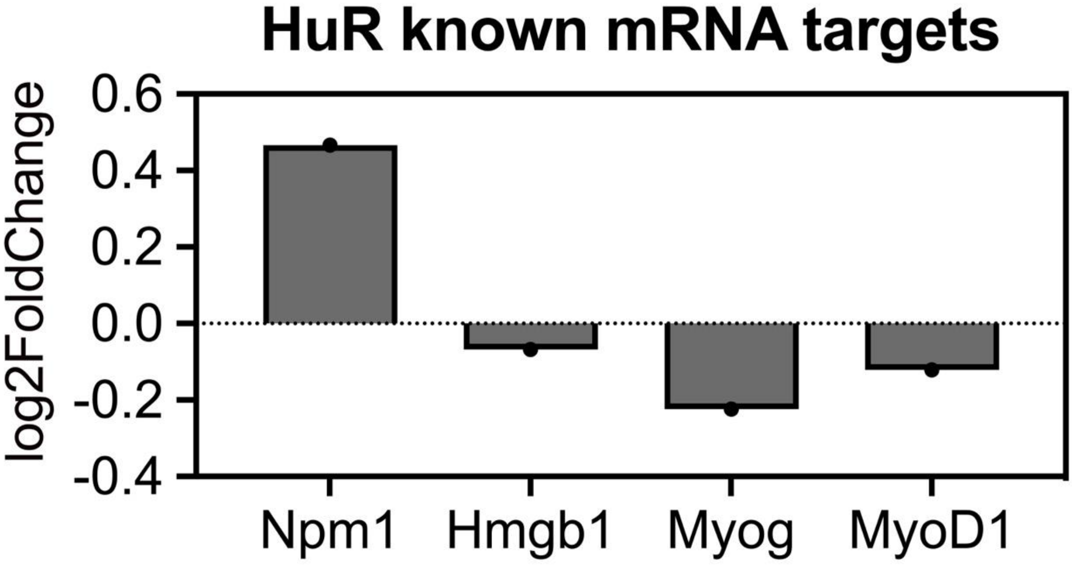

Fig. S4. HuR-known mRNA targets in muscle, related to Figure 4. Expression of known HuR mRNA targets from Differential expression analysis (DESseq2) by log2fold changes following RNA-sequencing of the tibialis anterior muscle of Elavl1$^{fl/fl}$ and Myf5-cre$^{/+}$Elavl1$^{fl/fl}$ mice. Analysis was done with n=3 mice per group.

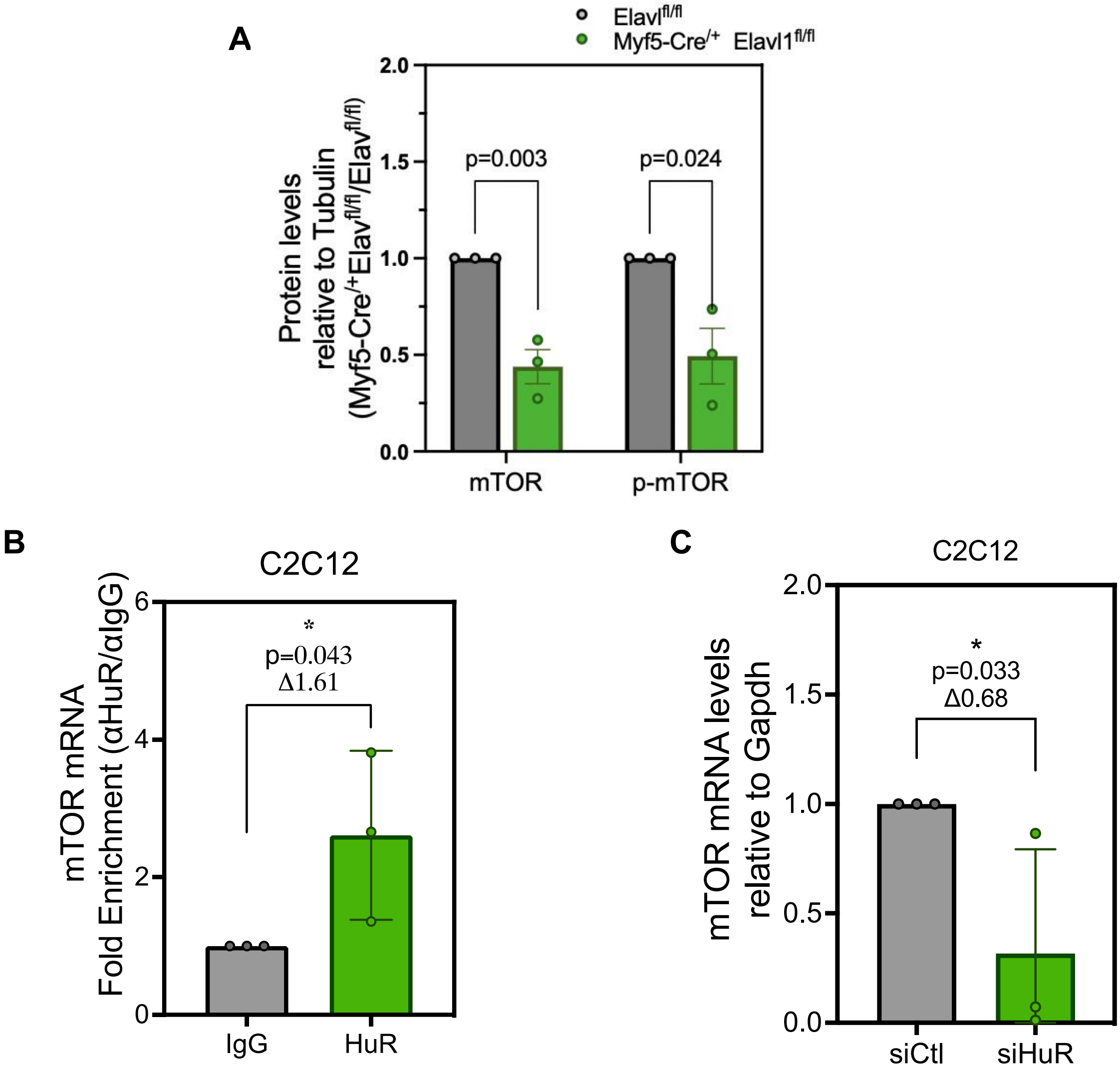

Fig. S5. HuR regulates mTOR expression, related to Figure 5.
(A) Quantification of mTOR protein and phosphorylation of mTOR at Ser2448 in Figure 5B normalized to Tubulin. (**B**) Fold enrichment of mTOR mRNA following immunoprecipitation of HuR normalized to IgG in C2C12 cells. **(C)** mTOR mRNA levels relative to GAPDH mRNA were measured by RT-qPCR in differentiated C2C12 cells depleted (siRNA) or not (siCtl) of HuR. The columns on the plots represent the mean with standard deviation, and unpaired t-test (*=p<0.05).

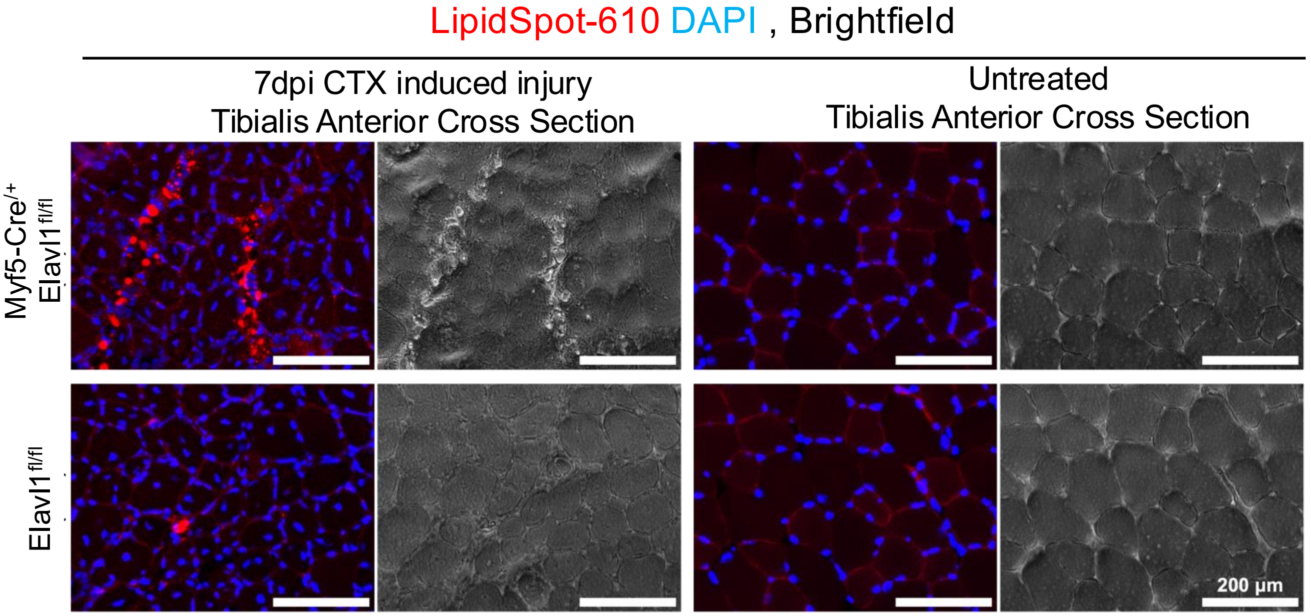

Fig. S6. The Myf5-Cre driven knockout of HuR induces an increase in lipid droplets during muscle regeneration.

Zoomed in image of Lipid droplets shown in Figure 6D using LipidSpot dye in tibialis anterior muscles obtained from CTX-injury (7 days-post injection) induced Elavl1$^{fl/fl}$ control and Myf5-cre$^{/+}$Elavl1$^{fl/fl}$ mice. Left: LipidSpot, Right: Brightfield. Scale =250μm.

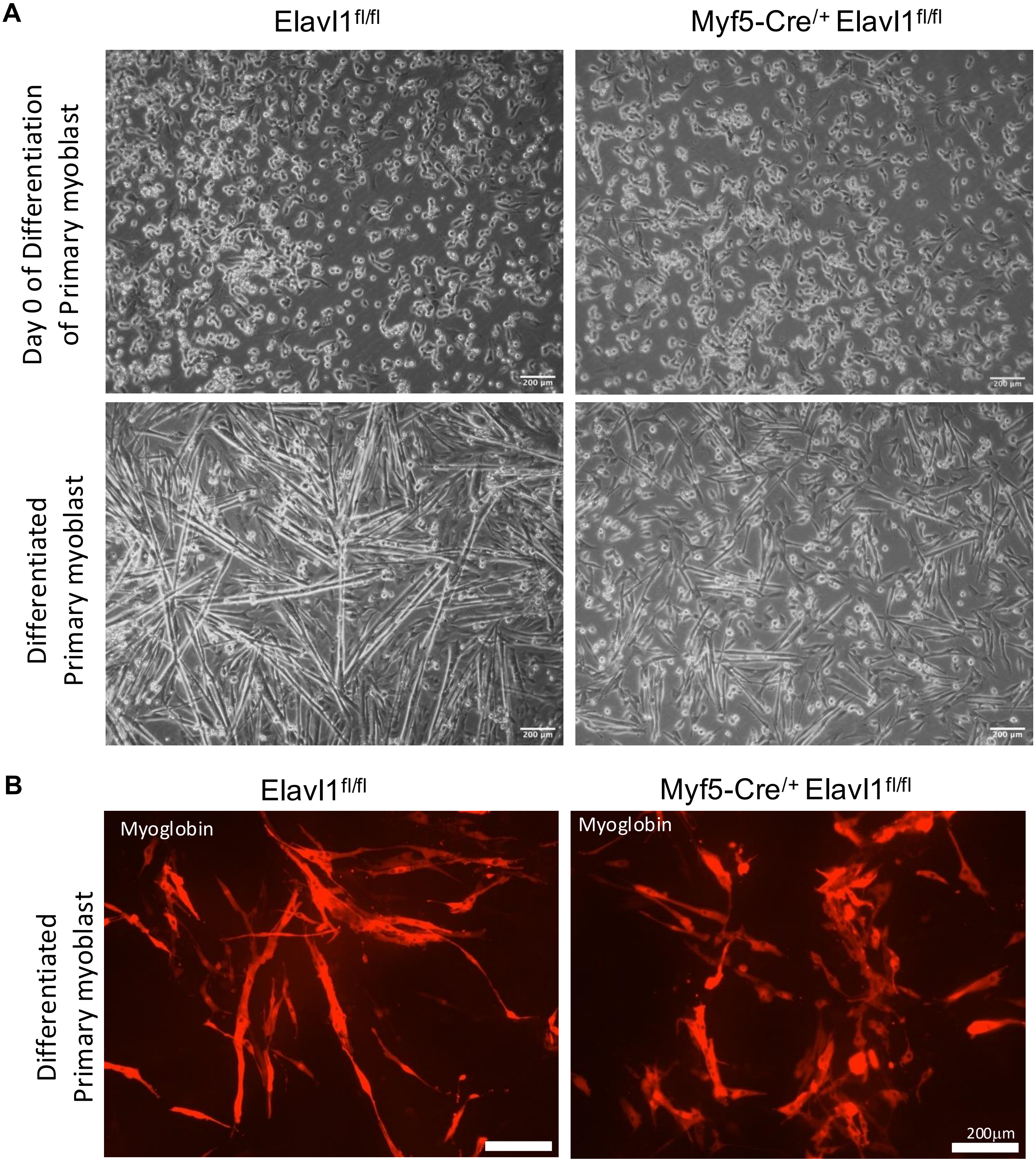

Fig. S7. Differentiated primary myoblast from the muscles of Elavl1fl/fl and Myf5-cre/+Elavl1fl/fl mice.

(A) Brightfield images of primary myoblast at day 0 of differentiation (Top) and differentiated primary myoblast (Bottom) from the muscles of Elavl1fl/fl and Myf5-cre/+Elavl1fl/fl mice. Cells of both groups were plated at 80% confluence before induction of differentiation. Scale =200µm. **(B)** Immunofluorescent images of differentiated primary myoblast labelled with terminal markers of muscle Myoglobin from the muscles of Elavl1fl/fl and Myf5-cre/+Elavl1fl/fl mice. Cells of both groups were plated at 80% confluence before induction of differentiation. Scale =200µm.

Key Resources Table

REAGENT or RESOURCE	SOURCE	IDENTIFIER
Antibodies		
HuR (3A2)	In house	N.A.
Tubulin	Developmental studies Hybridoma Bank	N.A.
Myosin heavy chain IIa	Developmental studies Hybridoma Bank	SC71
Myosin heavy chain IIb	Developmental studies Hybridoma Bank	BFF3
Pan-Laminin	Sigma-Aldrich	L9393
OXPHOS cocktail	Abcam	ab110413
Myosin heavy chain (embryonic)	Developmental studies Hybridoma Bank	F1.652
Pax7	Developmental studies Hybridoma Bank-	AB528428
Perilipin A/B	Sigma	P1873
MyHC (MF-20)	Developmental studies Hybridoma Bank	AB2147781
UCP1	Abcam	ab234430
mTOR	Cell Signaling	#2972

Phospho-mTOR (Ser2448)	Cell Signaling	#2971
OXPHOS complex II (SDHA)	Cell Signaling mAb	(#11998
Myoglobin	abcam	#11998
PGC-1α	Abcam	ab191838
Alexa Fluor 488 IgG_1	Invitrogen	#A21121
Alexa Fluor 555 IgM	Invitrogen	#A21426
Alexa Fluor 647 IgG	Invitrogen	#21244
F(ab')2-Goat anti-Rabbit IgG (H+L) Cross-Adsorbed Secondary Antibody, Alexa Fluor 488	Invitrogen	A-11070
Lipidspot-610	Biotium	70069T
Chemicals, peptides, and recombinant proteins		
Cardiotoxin	Cerdarlan	L8102
Actinomycin D (Act. D)	Sigma	A1410
Critical commercial assays		
miRCURY LNA miRNA PCR assay (200)	Quiagen	3621594
Experimental models: Cell lines		

C2C12 murine myoblast	ATCC, Manassas, VA, USA	N.A.
Primary myoblast	This paper	Myf5-Cre$^{/+}$Elavl1$^{fl/fl}$ and control Elavl1$^{fl/fl}$
Experimental models: Organisms/strains		
Myf5-Cre/+	(69)	N.A.
Elavl1$^{fl/fl}$	(61)	N.A.
Oligonucleotides		
Elavl1fl/fl-FW-5’-TGGTTATGAAGACCACATGGCGGAAGA-3’	(31)	N.A.
Elavl1fl/fl-REV-5’AGCTTAGCAGGTACCGTCTC-3’	(31)	N.A.
Cre-FW-5’-CATTTGGGCCAGCTAAACAT-3’	(31)	N.A.
Cre-REV-5’-CGGATCATCAGCTACACCAG-3’	(31)	N.A.
HuRexon2-FW-5’-ATATCATGTTCCCAACTCCC-3’	(31)	N.A.

HuRexon2-REV-5'-TGGCACTCACTGAACTGGAA-3'	(31)	N.A.
Myf5-Cre -FW-5'-CGTAGACGCCTGAAGAAGGTCAACCA-3'	JAX	stock #007845
Myf5-Cre -REW-5'-CACATTAGAAAACCTGCCAACACC-3'	JAX	stock #007845
Myf5-Cre-mutant-5'-ACGAAGTTATTAGGTCCCTCGAC-3'	JAX	stock #007845
miRNA target mouse gene: UniSp6	Qiagen	yp00203954
miRNA target mouse gene: hsa-miR-133b	Qiagen	yp00206058
miRNA target mouse gene: hsa-miR-133a-3p	Qiagen	yp00204788
Ctl	Ambion	siCtl: Scrabled,<br>-80uM (2 hits)
HuR	Ambion	siHuR: AM4390815 (s67964)<br>-80uM (2 hits)

RT-qPCR target gene (mouse)	Forward Primer sequence 5'-3'	Reverse Primer sequence 5'-3'
HuR-exon2	GGATGACATTGGGAGAACGA	CGTTCAGTGTGCTGATTGCT
GAPDH	AAGGTCATCCCAGAGCTGAA	AGGAGACAACCTGGTCCTCA
MyHC I	CTCAAGCTGCTCAGCAATCTATTT	GGAGCGCAAGTTTGTCATAAGT
MyHC IIA	AGGCGGCTGAGGAGCACGTA	GCGGCACAAGCAGCGTTGG
MyHC IIX	GAGGGACAGTTCATCGATAGCAA	GGGCCAACTTGTCATCTCTCAT
MyHC IIB	CACCTGGACGATGCTCTCAGA	GCTCTTGCTCGGCCACTCT
Myogenin	CTACAGGCCTTGCTCAGCTC	AGATTGTGGGCGTCTGTAGG
MyoD	CGACACCGCCTACTACAGTG	TTCTGTGTCGCTTAGGGATG
PDRM16	CAGCACGGTGAAGCCATTC	GCGTGCATCCGCTTGTG
UCP1	CGATGTCCATGTACACCAAGGA	ACCCGAGTCGCAGAAAAGAAG
Insig1	TTTGTGGTGGACATTTGATCGT	GCTAGGAAGGCGATGGTAATCC
ATGL	GACAGCTCCACCAACATCCA	GAGGCGGTAGAGATTGCGAA
mTOR	AGAAGGGTCTCCAAGGACGACT	GCAGGACACAAAGGCAGCATTG
Software and algorithms		

Graph pad prims	version 9.3.1	
Adobe illustrator	v.26.0.3.	
Image J fiji	used for manual accessment of H&E and immunofluoresecne image	https://imagej.net/software/fiji/downloads
ImageJ-MOSAICsuite	used for all H&E and immunofluoresecne image	https://imagej.net/plugins/mosaicsuite
ImageJ-Open CSAM	(Desgeorges et al., 2019)-used for Myf5xx-Regeneration	https://skeletalmusclejournal.biomedcentral.com/articles/10.1186/s13395-018-0186-6
MuscleJ	(Mayeuf-Louchart et al., 2018).-used for fiber type classification Myf5xx-TA	https://skeletalmusclejournal.biomedcentral.com/articles/10.1186/s13395-018-0171-0
BIOSEB software (BIO-CIS) for BIOSEB's Grip Strength Test (Model GT3)	Version 1.5.1.0 (26-10-2018)	https://www.bioseb.com/en/activity-motor-control-coordination/48-grip-strength-test.html
SEDACOM Software for Five Lanes Touchscreen Convertible Treadmill, Mice	V2.0	https://www.harvardapparatus.com/sedacom-software.html
Specialized equipment		
Hamilton microsyringe	Sigma	#20734
BIOSEB's Grip Strength Test	Bioseb	Model GT3

Touchscreen Treadmill (Panlab)	Harvard apparatus (panlab)	Model LE8710MTS / serialN 2827219
qPCR apparatus	MBI lab equipment	Corbett Research RG-6000
Cryostat	Leica model S/N: 1436/10.2000	1900-03-01
Cryostat	HM 525	Thermo
Reagents		
2-Methylbutane	Sigma	M32631-500ml
Tragacanth powder (100g)	AlfaAesar	A18502
VWR clearfrozen section compound	VWR	95057-838
VECTASHIELD® HardSet™ Antifade Mounting Medium with DAPI	Vector labs	H-1500-10
M.O.M Blocking reagent	Vectors Lab	MKB-2213
LS Colums	miltenyibiotech	130-042-401
steriflip	millipore	SCNY00100
DMEM+Glutamax	Gibco	31966
F12-Glutamax	Gibco	31765
FBS	SIgma	F1051
Ultroger G	Cederlane	15950-017

DPBS	Sigma	D8537
P/S	sigma	P0781-100ml
Trypsin (no red, for satellite cell)	Life Tech	15090-056
Trypsin (regular tissue culture)	Sigma	T4049-500ml
Collagenase D	Sigma-Roche	1E+10
Red Bloood Cell Lysis buffer	Sigma	R7757-100ml
Anti-Integrin a7 beads	Miltenyi	130-104-401
Satelllite cell isolation kit mouse	Miltenyi	130-104-268
QuadroMACS™ Separator	miltenyibiotec	130-090-976
Macs multistand	miltenyibiotec	14750
Trizol Reagent	Ambion	2E+07
iScript™ Reverse Transcription Supermix	Biorad	2E+06
jetPRIME	Polyplus transfection reagent	101000027
Sso Fast EvaGreen ® Supermix	BioRad	#1725204
